# Human ACE2 expression, a major tropism determinant for SARS-CoV-2, is regulated by upstream and intragenic elements

**DOI:** 10.1371/journal.ppat.1011168

**Published:** 2023-02-22

**Authors:** John N. Snouwaert, Leigh A. Jania, Trang Nguyen, David R. Martinez, Alexandra Schäfer, Nicholas J. Catanzaro, Kendra L. Gully, Ralph S. Baric, Mark Heise, Martin T. Ferris, Elizabeth Anderson, Katia Pressey, Jacob A. Dillard, Sharon Taft-Benz, Victoria K. Baxter, Jenny P-Y Ting, Beverly H. Koller

**Affiliations:** 1 Department of Genetics, University of North Carolina at Chapel Hill, Chapel Hill, North Carolina, United States of America; 2 Lineberger Comprehensive Cancer Center, University of North Carolina, Chapel Hill, North Carolina, United States of America; 3 Department of Epidemiology, Gillings School of Global Public Health, University of North Carolina at Chapel Hill, Chapel Hill, North Carolina, United States of America; 4 Department of Microbiology and Immunology, School of Medicine, University of North Carolina at Chapel Hill, Chapel Hill, North Carolina, United States of America; 5 Department of Pathology and Laboratory Medicine, University of North Carolina at Chapel Hill, Chapel Hill, North Carolina, United States of America; 6 Center for Translational Immunology, University of North Carolina at Chapel Hill, Chapel Hill, North Carolina, United States of America; The Ohio State University, UNITED STATES

## Abstract

Angiotensin-converting enzyme 2 (ACE2), part of the renin-angiotensin system (RAS), serves as an entry point for SARS-CoV-2, leading to viral proliferation in permissive cell types. Using mouse lines in which the Ace2 locus has been humanized by syntenic replacement, we show that regulation of basal and interferon induced ACE2 expression, relative expression levels of different ACE2 transcripts, and sexual dimorphism in ACE2 expression are unique to each species, differ between tissues, and are determined by both intragenic and upstream promoter elements. Our results indicate that the higher levels of expression of ACE2 observed in the lungs of mice relative to humans may reflect the fact that the mouse promoter drives expression of ACE2 in populous airway club cells while the human promoter drives expression in alveolar type 2 (AT2) cells. In contrast to transgenic mice in which human ACE2 is expressed in ciliated cells under the control of the human FOXJ1 promoter, mice expressing ACE2 in club cells under the control of the endogenous Ace2 promoter show a robust immune response after infection with SARS-CoV-2, leading to rapid clearance of the virus. This supports a model in which differential expression of ACE2 determines which cell types in the lung are infected, and this in turn modulates the host response and outcome of COVID-19.

## Author summary

SARS-CoV-2, the virus responsible for COVID-19, infects the human respiratory tract by binding to angiotensin-converting enzyme 2 (ACE2), a protein on the outer surface of cells that is exposed to the air we inhale. Differences in the structure of ACE2 between mouse and man prevent the virus from infecting cells lining the airways of the mouse, limiting the usefulness of wildtype mice as a model system for studying COVID-19. To circumvent this problem, we have created two mouse lines in which the chromosomal segment that encodes the mouse ACE2 protein has been replaced with the equivalent segment of human DNA. In the first of these lines, expression of human ACE2 is regulated by the mouse upstream promoter, while in the second line it is regulated by the human upstream promoter. Using these mice, we show both qualitative and quantitative differences between ACE2 expression driven by the mouse and human promoters that are likely to impact disease progression in the two species. Mice expressing the full-length human ACE2 gene under the control of its own promoter should provide a useful model system for understanding the complex pathological processes and immune responses associated with COVID-19 in humans.

## Introduction

SARS-CoV-2 is capable of entering cells by two distinct mechanisms: through endocytosis into endosomes and through the direct fusion of the viral particle with the cell membrane [1,2]. Non-specific endocytosis followed by endosome lysosome maturation and activation of the spike protein by cathepsin L can results in viral production by highly endocytic cells [3]. On the other hand, direct infection of cells with coronaviruses by delivery of the SARS-CoV-2 RNA across the plasma membrane requires binding of the viral spike protein to a cell surface receptor. To date, ACE2 has been shown to serve as this receptor for three coronaviruses: NL63, SARS-CoV, and SARS-CoV-2 [4–6]. Fusion of the viral and host membranes is dependent both on activation of the spike protein by host proteases such as the type II transmembrane serine protease, TMPRSS2, and on juxtamembranous cleavage of ACE2 by TMPRSS2 [7,8]. While the relative role of these two mechanisms of viral entry into host cells at various stages of COVID pathogenesis has not been defined *in vivo*, it is generally agreed that initial infection is dependent on the expression of ACE2 by airway epithelial cells.

ACE2 is a type-1 transmembrane protein that includes extracellular monocarboxypeptidase and collectrin domains as a result of the fusion of an angiotensin-converting enzyme-like gene with a collectrin-like gene early in evolution [9,10]. Consistent with the presence of these very different extracellular domains, ACE2 has been assigned two different functions. In both humans and mice, ACE2 is most highly expressed in the gastrointestinal tract, where ACE2 functions as an accessory protein. In addition to a proposed role for ACE2 in digestion of peptides present in chyme in preparation for their transport, its collectrin domain ensures proper trafficking of the amino acid transporter, B^0^AT1 (*SLC6A19*), to the plasma membrane of enterocytes [11]. Deficiency in B^0^AT1 transport leads to reductions in tryptophan and glycine in the blood and to an inflammatory bowel disease [12–14]. However, it is the mono-carboxypeptidase activity of ACE2 that has been the focus of the vast majority of studies of ACE2 function.

The carboxypeptidase activity of ACE2 degrades the ACE-derived vasoconstriction peptide, angiotensin II (Ang II), in an organ specific manner, yielding the vasodilator peptide, Ang1-7 [15,16]. ACE2 is thus considered a key regulator of the renin-angiotensin system (RAS) [17]. A body of evidence suggests that this ACE2 enzymatic activity serves to counteract effects mediated by angiotensin converting enzyme (ACE), and it has been proposed that imbalances in ACE/ACE2 activity contribute to diseases such as hypertension, progressive renal disease, and diabetes, all of which have been associated with risk for severe COVID-19 [18]. In addition, a number of studies using *Ace2-/-* mouse lines support a role for ACE2 in protection from lung injury, perhaps independent of its contribution to Ang II homeostasis [19,20]. ACE2 displays activity towards a number of peptides in addition to Ang II that could contribute to a potential protective function in the lung and other organ systems. For instance, ACE2, along with ACE, has been assigned a regulatory role in the kallikrien-kinin pathway [21,22]. The prominent role of ACE2 in many of the physiological systems altered in COVID-19, including the multifocal tissue damage in the microcirculatory environment of many organs, supports the argument that SARS-CoV-2 activity provokes a transient molecular disease resulting in part from reduced ACE2 activity [23,24]. Changes in ACE2 activity after SARS-CoV-2 infection may be particularly problematic in individuals in whom ACE2 expression is already altered secondary to obesity, diabetes and hypertension [25,26].

A number of model systems have been developed for identifying the mechanisms underlying the regulation of *ACE2* expression as well as for elucidating the role that ACE2 plays in various physiological processes, including SARS-CoV-2 infection and COVID-19-associated pathology [27–31]. However, because these systems have been developed with the primary goal of obtaining sufficiently high ACE2 expression in the airways to allow SARS-CoV-2 infection, they may display developmental and tissue specific patterns of ACE2 expression that differ in important ways from those observed in humans. These differences in expression necessarily limit the usefulness of these systems in identifying and understanding the more subtle roles played by ACE2 in the development and resolution of COVID-19. To overcome these limitations, we have developed two mouse lines in which the ACE2 locus has been humanized by syntenic replacement. Characterization of ACE2 expression in these lines reveals that the mechanisms underlying the observed patterns of expression are unique to each species, differ between tissues, and are determined by both intragenic and upstream promoter elements. Furthermore, we show that differences in expression impact susceptibility to SARS-CoV-2 infection and immune responses.

## Results

### Generation of the humanized ACE2 lines

**Fig 1:**
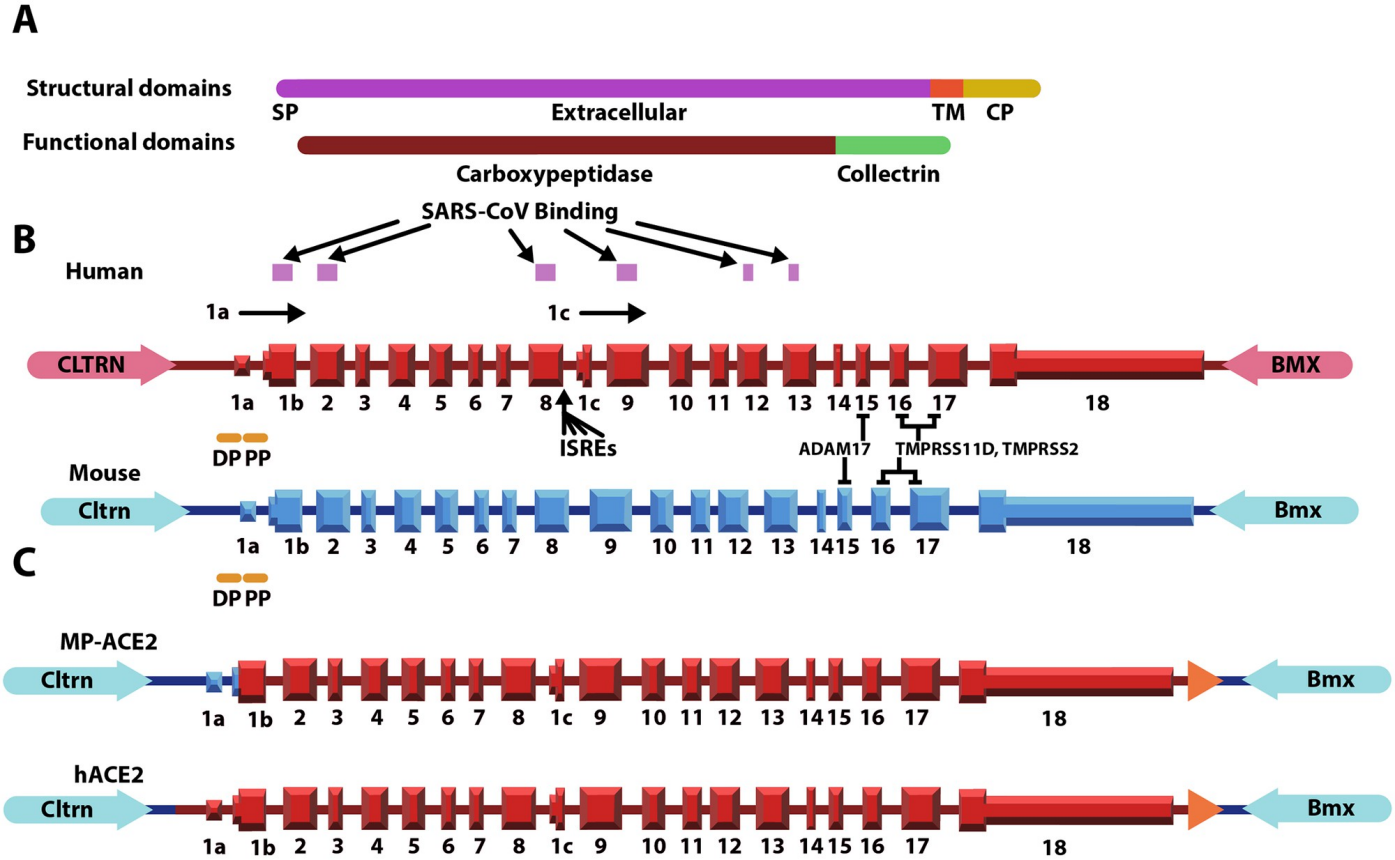
Schematic showing the protein domains and exon intron organization of the mouse and human ACE2 locus and the structure of the loci in the two humanized mouse lines. **A**. Protein structural and functional domains of the ACE2 protein. The ACE2 protein consists of a signal peptide (SP), an extracellular domain, a transmembrane domain (TM) and a cytoplasmic domain (CP). B. Exonic structure of human *ACE2* gene (red) and flanking genes (pink) and mouse *Ace2* gene (blue) and flanking genes (light blue). Regions of the human protein identified as interacting with the spike proteins of SARS-Co-V and SARS-CoV-2 are indicated by pink boxes above the human gene. Mouse ACE2 does not bind the spike protein in these two coronaviruses. The cleavage site for ADAM17 and TMPRSS11D/TMPRSS2 are shown. Tall boxes represent coding exons while shorter boxes represent 5’ and 3’ UTRs. In both species the start codon is located in the second exon, labeled here as 1b. Both mouse and human also express a transcript (not shown) which, although it lacks exon 1a, still gives rise to an ACE2 protein with the identical amino acid sequence. The distal promoter region (DP) and proximal promoter region (PP) are present in both mouse and human. The human gene contains a third transcriptional start site between exons 8 and 9, which gives rise to a shorter transcript encoding a protein expected to lack enzymatic function and the ability to bind SARS-CoV-2. Four interferon response elements (ISREs) are located in the promoter region upstream of the sequence that encodes the first exon of the shorter transcript (exon 1c). Exon 1c is only present in primates. C. Structure of humanized *Ace2* locus. The locus in which only the coding sequence is humanized, MP-ACE2, is shown above. In this locus the human sequence extends from the start codon to approximately 4 kb downstream of the 3’ end of the final exon. In the locus in which humanization includes the promoter, hACE2, the human sequence extends from approximately 3.5 kb upstream of the start codon to approximately 4 kb downstream of the 3’ end of the final exon. The orange arrow in each humanized locus represents the floxed neomycin resistance marker, which is removed by transient expression of Cre.

The mouse and human *Ace2/ACE2* genes are located on the X chromosome in the region neighboring the pseudoautosomal region [32]. The mouse and human genes are remarkably similar, both in their structure and in the protein domains they encode (Fig 1A and 1B). The ectodomain of both proteins can be cleaved by ADAM17 to generate soluble ACE2 (sACE2) [8]. Differences have been noted in the regulatory elements that control expression of the mouse and human genes, with the most notable being the presence of an intragenic promoter upstream of a primate-specific exon within intron 8 of the human gene. Initiation of transcription from this internal promoter gives rise to a transcript encoding a truncated isoform of *ACE2*, designated *dACE2* or short ACE2 [33]. The encoded protein lacks both the enzymatic activity of the full-length protein and its ability to bind SARS-Cov-2. Both human and mouse ACE2 can catalyze the formation of Ang1-7 from Ang II [34], although some differences have been noted in their enzymatic activity.

**Fig 2:**
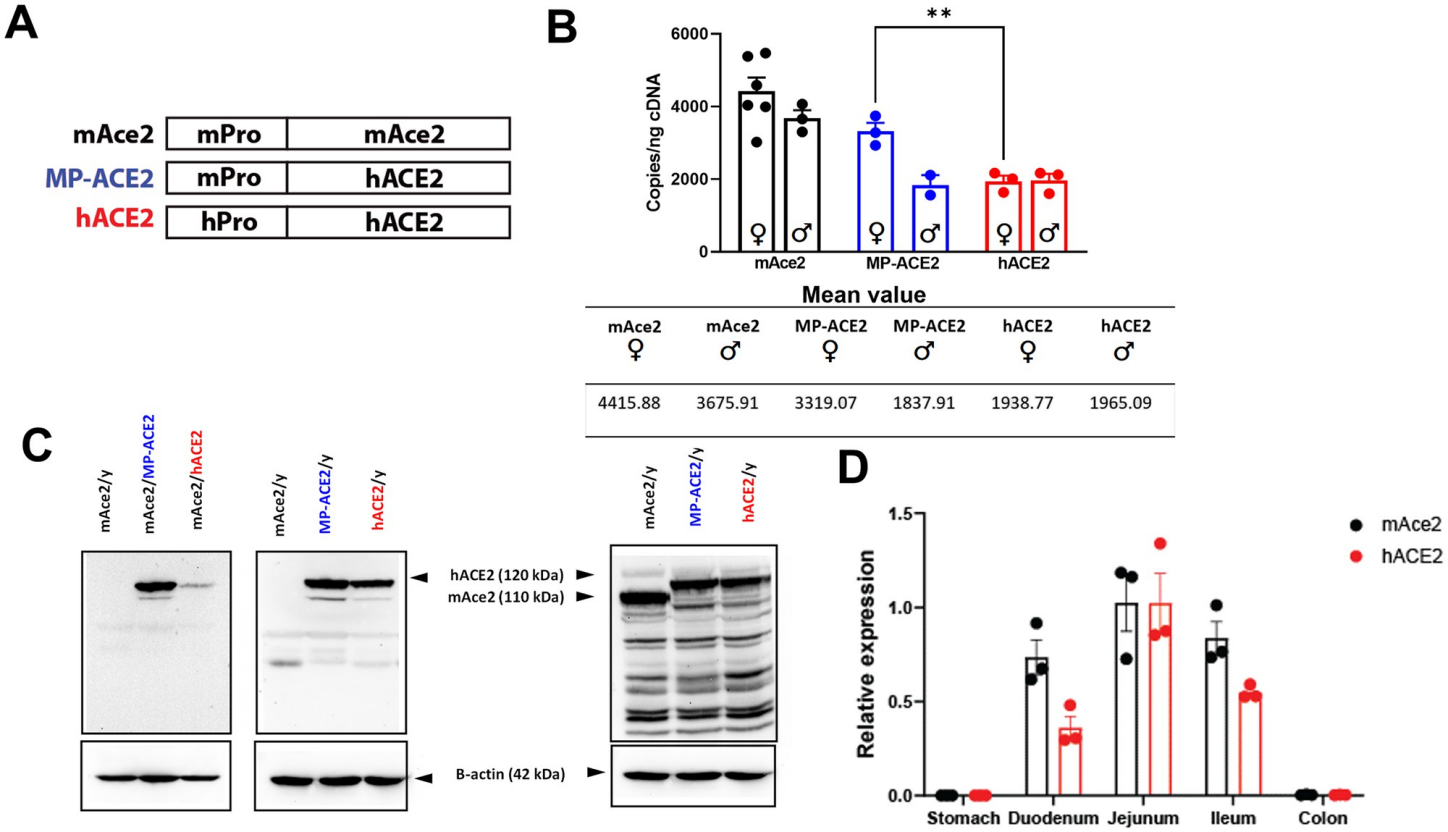
Comparison of *ACE2* expression in intestinal tissue of the humanized mouse lines. **A**. Ideogram of the structure promoter/exon intron origin, and nomenclature/color scheme used in referring to the three mouse lines compared in these studies. mAce2 has the mouse promoter and coding sequence, MP-ACE2 has the mouse promoter but human coding sequence, and hACE2 has the human promoter and coding sequence. **B**. Droplet digital PCR (ddPCR) evaluation of ACE2 expression in cDNA prepared from male (♂) and female (♀) mice of the indicated genotype. Mean value for each is shown below. **C**. Western analysis of lysates prepared from mice of the indicted genotype/sex with the human-specific MAB933 antibody and ab15348, which recognizes both human and mouse ACE2. **D**. Comparison of the proximal to distal expression in the intestinal tract of *ACE2/Ace2* in female mice heterozygous for the mouse locus (mACE2) and humanized locus (hACE2). Expression observed in the jejunum for the human and mouse gene was assigned a value of 1. ** p<0.01. For statistical comparison between all groups in **B**, see S1 Table.

We used syntenic replacement of the endogenous mouse *Ace2* gene to generate two mouse lines that are expected to express human *ACE2* (Fig 1C). The two lines differ only in the location of the 5’ crossover event, with MP-ACE2 leaving the mouse promoter and 5’ UTR in place, while hACE2 replaces the entire *Ace2* gene, including 5’ regulatory regions, with a 51 kb syntenic segment of the human locus. This includes 3.5 kb of sequence upstream of the start codon. An ideogram of the structure of the *ACE2* locus present in the mouse lines included in this study along with nomenclature used in referring to them is shown in Fig 2A.

In defining the transition sites from mouse to human DNA in the two lines, we considered information available concerning the promoter of the mouse and human genes and presence of transcription factor binding motifs [35]. For example, two evolutionarily conserved regions, referred to as the distal and proximal promoter regions, have been identified just upstream of the *ACE2* translational start codon in both the mouse and human genes [36]. Mice and humans both express *ACE2* transcripts that include an untranslated first exon (exon 1a) [37,38]. In the MP-ACE2 mouse line, the mouse/human transition occurs at the translational start site in exon1b, thus maximizing the length of the upstream mouse regulatory region and ensuring that splicing events involved in generation of the 5’UTR occur between mouse-derived sequences.

### Expression of ACE2 in intestinal tract of humanized mice

*ACE2/Ace2* is highly expressed in both the human and mouse intestinal tract, where it is located at the luminal membrane of small intestine enterocytes. Expression is also observed, albeit at lower levels, in intestinal crypt cells and in the colon [12]. We initiated characterization of the humanized mouse lines by examination of transcript levels in the jejunum (Fig 2 and S1 Fig). *ACE2* is located on the X chromosome in both humans and mice. If X inactivation of the locus is random, approximately 50% of the cells in tissues from female offspring will express the human gene and approximately 50% will express the mouse gene. To simplify comparison of *ACE2* and *Ace2* expression between female and male mice throughout this report, the number of copies of *Ace2* and *ACE2* transcripts present in cDNA prepared from females heterozygous for the humanized locus (mAce2 x MP-ACE2 or mAce2 x hACE2) were multiplied by two.

Analysis of mRNA from the jejunum showed expression of *Ace2 and ACE2* in the mAce2 and MP-ACE female mice, with mean values of 4415 and 3319 copies per ng of cDNA respectively (Fig 2B). Expression of *ACE2* in the two MP-ACE2 males was slightly lower than *Ace2* expression in mAce2 males (Fig 2B). This was surprising as, while sexual dimorphism for ACE2 has been reported, for the majority of tissues examined ACE2 is reported to be expressed at higher levels in males [39,40]. The expression of *ACE2* in the mouse line carrying the fully humanized locus (hACE2) was also reduced by about 50% compared to mACE2 (Fig 2B). However, in this case no sexual dimorphism was observed. The human promoter drove reduced gene expression of ACE2 in both hACE2 males and females compared to their mAce2 count parts.

We determined if the difference we noted in *ACE2* mRNA levels between lines correlated with differences in protein levels detectable by western analysis (Fig 2C). ACE2 levels were measured in lysates prepared from the jejunum of mAce2, MP-ACE2 and hACE2 mice using two different anti-ACE2 antibodies, MAB933 and ab15348. MAB933 is a species specific monoclonal antibody that recognizes the extracellular region of human ACE2, and ab15348 is a rabbit serum raised against epitopes present in the intracellular/carboxyl terminal domain of mouse and human ACE2. The anti-ACE2 monoclonal, MAB933, detected a 120 kDa protein in lysates prepared from male and female MP-ACE2 and hACE2 animals. As expected, the MAB933 did not identify any band corresponding to ACE2 in lysates prepared from the mAce2 mice. Consistent with the lower mRNA levels of *ACE2* in female hACE2 mice compared to MP-ACE2 females, a noticeably less intense 120 kDa band was observed in the females carrying the fully humanized line.

Expression of ACE2 was then compared in lysates from the jejunum of mAce2, MP-ACE2 and hACE2 male mice. Consistent with the similar mRNA levels, the human specific antibody, MAB933, detected a 120 kDa band of similar intensity in the lysates from the MP-ACE2 and hACE2 mice, while no signal was observed in the lane corresponding to the sample from mAce2 animals (Fig 2C).

Lysates were also analyzed with anti-ACE2 serum, ab15348, which recognizes both human and mouse ACE2 proteins (Fig 2C). Similar to previous reports, we noted the differing mobility of mouse ACE2 and human ACE2 on western analysis [41,42]. Although human and mouse ACE2 are 805 a.a. in length (92,463 Da) and the predicted molecular weight of ACE2 from the two species is virtually identical, mouse ACE2 consistently displayed a lower molecular weight on western analysis than the human protein, perhaps reflecting the absence of two of the seven N-glycosylation sites present in human ACE2 [43]. Also apparent on analysis of the lysates with MAB933 and ab15348 were differences between these antibodies in there cross reactivity with other proteins present in the lysates. Despite this limitation, the ab15348 anti-ACE2 antibody identified ACE2 protein in lysates from both species, with the highest intensity corresponding to lysates prepared from the jejunum of the mAce2 mice. This is consistent with the 2 fold higher *Ace2* cDNA copies in male mAce2 mice when compared to the two humanized lines (Fig 2B). Despite differences in the level of expression, human and mouse *ACE2/Ace2* showed a remarkably similar pattern of expression, with the lowest levels observed in the stomach and colon (Fig 2D).

### ACE2 expression in kidney and heart

**Fig 3:**
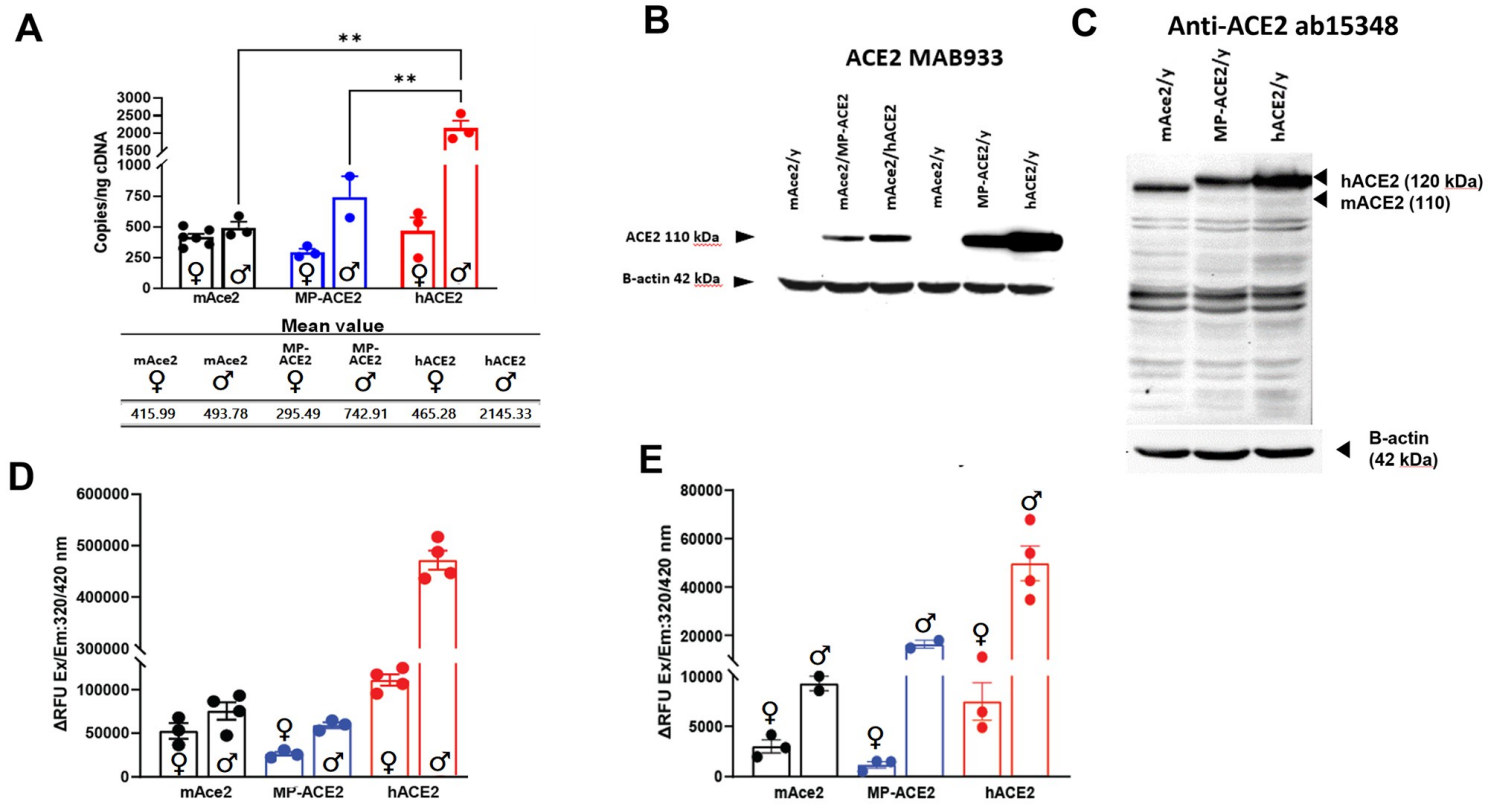
Comparison of expression of *ACE2* in kidney of the humanized mouse lines. **A**. Droplet digital PCR (ddPCR) evaluation of cDNA prepared from male (♂) and female (♀) mice of the indicated genotype. Mean value for each is shown below the bar graph. **B&C**. Western analysis of lysates prepared from mice of the indicted genotype and sex with the human specific MAB933 antibody **(B)** and ab15348 **(C)**, which recognizes both human and mouse ACE2. **D**. kidney ACE2 carboxypeptidase activity measured at one hour after initiation of the reaction. **E**. ACE2 carboxypeptidase activity in urine collected from mice of indicated sex and genotype determined one hour after initiation of the reaction. ** p<0.01. For statistical comparison between all groups in **A** see S1 Table. For statistical comparison between all groups in **D** and **E** see S1 Table.

Both human and mouse express high levels of *ACE2/Ace2* in the kidney, and approximately 50% of *ACE2* expression is associated with proximal tubule cells, where it is localized to the apical (luminal) brush border of the epithelial cells [44,45]. We examined *ACE2/Ace2* mRNA levels in mAce2, MP-ACE2, and hACE2 mice by ddPCR. As predicted by previous studies, expression of *Ace2* in the kidney of the mAce2 mice was lower than that observed in the intestinal tract, with approximately 500 copies/ng of RNA compared to 4,000 copies/ng in the intestinal tract (Compare Figs 2B and 3A). Consistent with previous reports, expression of *Ace2* was slightly higher in males [46], but in our evaluation by ddPCR, this difference failed to reach significance. Robust expression of *ACE2* was observed in the MP-ACE2 females; however, levels were significantly lower than those measured in mAce2 animals. This decrease in *ACE2* expression was not observed in males. In contrast, although only two MP-ACE2 males were included in the study, expression of *ACE2* in these animals was similar to that observed in mAce2 males, suggesting that regulatory elements contributing to sexual dimorphism in *ACE2* expression may reside within the human coding segment of the ACE2 locus. However, a prominent role for the promoter in driving sexual dimorphism was supported by our examination of expression of *ACE2* in mice with the fully humanized locus, as a four-fold increase in *ACE2* transcripts was observed in male hACE2 kidneys compared to those from females (Fig 3A).

We determined whether these differences in mRNA levels translated into differences in ACE2 protein levels in the kidney. Western blot analysis using the human specific anti-ACE2 antibody MAB933 of lysates prepared from MP-ACE2 and hACE2 mice showed an increase in protein levels in the kidney of the mice expressing the fully humanized locus. Consistent with the small increase in mRNA levels in the female hACE2 mice compared to their MP-ACE2 counterparts, an increase in ACE2 was observed in lysates from the females carrying the fully humanized locus (Fig 3B).

We next compared ACE2 protein levels in kidney lysates prepared from mAce2, MP-ACE2 and hACE2 males using the rabbit polyclonal antibody ab15348, which recognizes both human and mouse ACE2 (Fig 3C). The hierarchy of expression of *ACE2/Ace2* mRNA in the three mouse lines was recapitulated on western blot analysis, with the lowest levels of expression observed in mAce2 mice, a slight increase observed in MP-ACE2 line, and a dramatic increase in the hACE2 kidney.

Together, our results suggest that elements within the human promoter amplify sexual dimorphism in ACE2 expression in the kidney. However, minor contribution by elements within the coding regions of the gene is also supported by the less dramatic difference we observed between the sexes in ACE2 expression in the MP-ACE2 mice compared to hACE2 mice.

Similar studies of expression of ACE2 in the mouse heart were carried out. Again, a very complex pattern of expression was observed, with the mouse promoter failing to drive levels of ACE2 expression observed in wild type mice. The lower levels of expression in the MP-ACE2 mice compared to the hACE2 mice suggested interaction between intergenic and upstream regulatory regions (S2 Fig).

### Evaluation of ACE2 carboxypeptidase activity in humanized mouse lines

*ACE2* encodes a carboxypeptidase, and thus enzymatic activity is commonly used as a surrogate measure of ACE2 protein levels [47]. Activity towards a known ACE2 substrate is assessed by carrying out the reaction in the presence and absence of an ACE2 inhibitor [41]. An important advantage of this method is the ability to measure soluble ACE2 (sACE2). The ectodomain of ACE2 is shed from the cell membrane [48], both constitutively and in response to physiological changes; however, sACE2 retains both its carboxypeptidase activity and ability to bind SARS-CoV-2 [49]. We therefore examined ACE2 activity in both kidney lysates and urine (Fig 3D and 3E). sACE2 was detectable in urine [50], likely reflecting shedding from epithelia of the proximal tubules [51], as its size makes it unlikely that it is excluded from the glomerular filtrate. ACE2 activity in both the kidney and the urine from the mAce2, MP-ACE2 and hACE2 mouse lines generally aligned with the number of *Ace2/ACE2* transcripts observed by ddPCR. Sexual dimorphism was observed in all three lines, but the magnitude of the difference between male and females was increased with humanization of the locus.

### ACE2 expression in the lung and airways

The central role of the lungs in COVID-19 related morbidity and mortality is surprising given the extremely low level of expression of this gene in the human lung, with a normalized expression level of 0.8 compared to 122.0 for the small intestine, 23.0 for the kidney, and 10.5 for the heart [52,53]. In contrast, a comparison of ACE2 activity between mouse organs reported that ACE2 activity in the mouse lung was relatively high, being approximately 25% that of the kidney, and no significant sex bias was observed [46]. We therefore determined whether this species difference in *ACE2/Ace2* expression in the airways was reiterated in the two humanized mouse lines.

**Fig 4:**
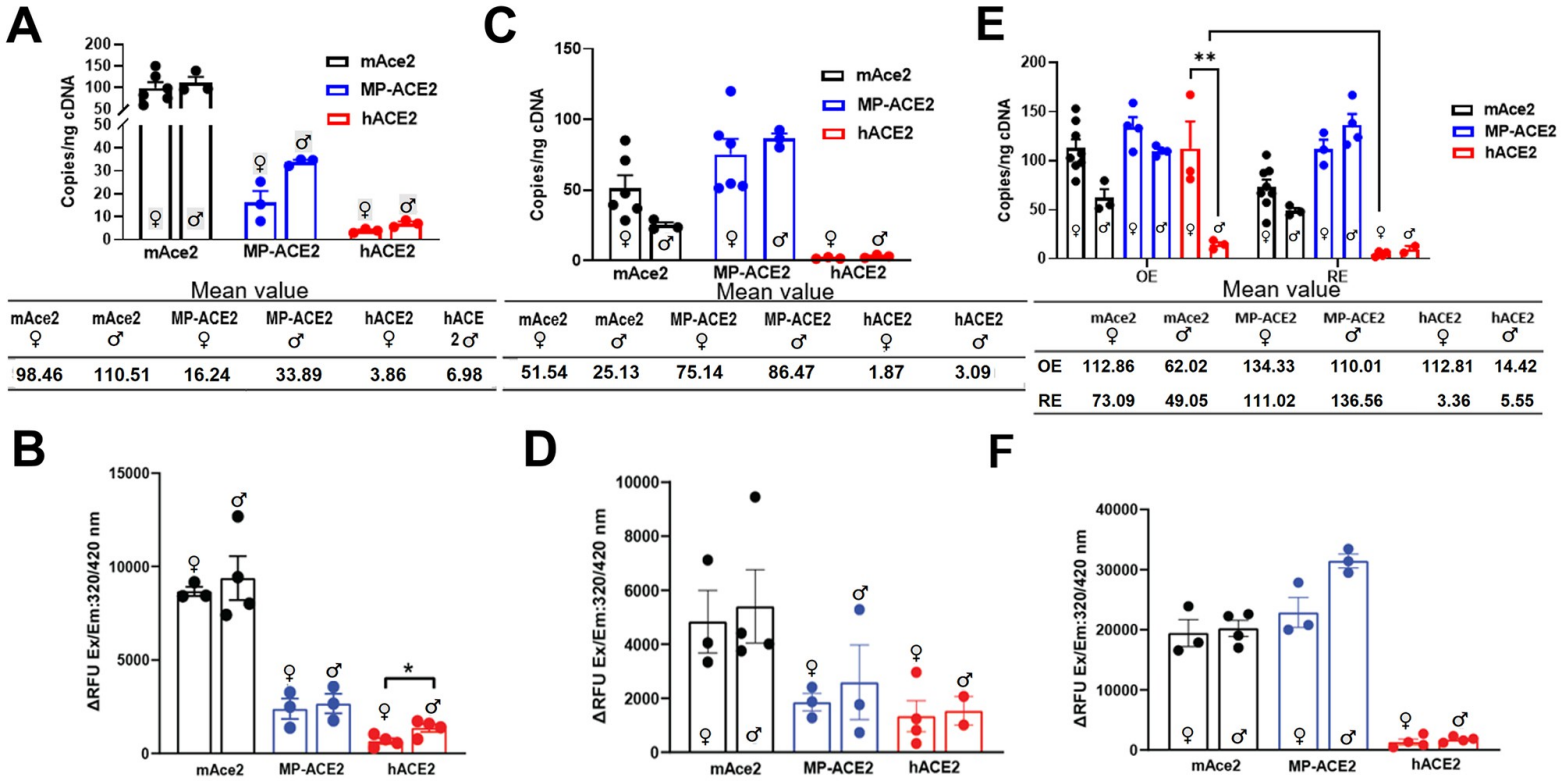
Comparison of ACE2 expression in lung and airways of humanized mouse lines. **A**,**C**,**E**. ddPCR evaluation of cDNA prepared from male (♂) and female (♀): **A**, lung; **C**, trachea; **E**, nasal epithelium of mice of the indicated genotype. Mean number of copies per nanogram of cDNA for each group is shown in the table below the bar graphs for each tissue. **B**, **D**, **F**. ACE2 carboxypeptidase activity in samples evaluated for expression: **B**, activity in lung; **D**, activity in trachea; **F**, activity in nasal epithelium (respiratory). Activity in each sample was measured 1 hour after initiation of the reaction. For group sizes and statistical comparison between all groups in **A**, **C**, and **E**, see S1 Table. For group sizes and statistical comparison between all groups in **B**, **D**, and **F**, see S2 Table. OE, olfactory epithelium; RE, respiratory epithelium.

Lung, trachea, nasal epithelia were collected from mAce2, MP-ACE2, and hACE2 mice and evaluated for expression of *ACE2/Ace2* by ddPCR (Fig 4A, 4C and 4E). In collection of the nasal epithelia, the respiratory and olfactory epithelia were isolated individually from the three mouse lines for mRNA expression analysis (Fig 4E). The enrichment for olfactory and respiratory epithelium was evaluated by examining expression of genes characteristic of these two tissues: *Muc5a* for respiratory epithelium and *Ugt2a1/2* for olfactory epithelium (S3 Fig) [54,55]. As an additional indicator of levels of ACE2 protein levels, carboxypeptidase activity was determined in tissue homogenates for all tissues except olfactory epithelium (Fig 4B, 4D and 4F).

Robust expression of endogenous murine *Ace2* gene was seen in the lung, with over 100 copies/ng cDNA, only four-fold lower than that observed in the kidney (~400 copies/ng). ACE2 carboxypeptidase activity paralleled this high expression, with activity approximately 25% of that observed in the mAce2 kidney (Figs 3D vs 4B). In contrast, expression of the humanized *ACE2* locus in the hACE2 lung was approximately 100 fold lower than in the kidney, in agreement with the relative expression of *ACE2* in these two organs in humans [52]. Slightly higher *ACE2* expression was seen in the lung, trachea and nasal respiratory epithelia of male hACE2 mice relative to females, although this difference did not reach the level of statistical difference. In contrast, *ACE2* expression in the olfactory epithelium of female hACE2 mice was approximately eight-fold higher than that measured in male hACE2 mice and more than 15-fold higher than that measured in other parts of the airway for either sex.

Expression in the airways of the MP-ACE2 mice was generally higher than that from the endogenous mouse *Ace2* locus, except in the lungs, where expression from the *MP-ACE2* locus was reduced by approximately 80% in female and 60% in male lungs relative to expression from the *Ace2* locus (Fig 4A). These results were somewhat unexpected, since expression from both loci is driven by the same endogenous mouse upstream promoter elements. ACE2 specific carboxypeptidase activity was easily measurable in the airway epithelia of the MP-ACE2 and hACE2 mice, verifying translation of the human transcripts (Fig 4B, 4D and 4F). The decrease in MP-ACE2 expression in the lung was paralleled by an approximate fourfold decrease in human ACE2 protein levels (Fig 4B).

### ACE2 expression in lung club and AT2 cells

**Fig 5:**
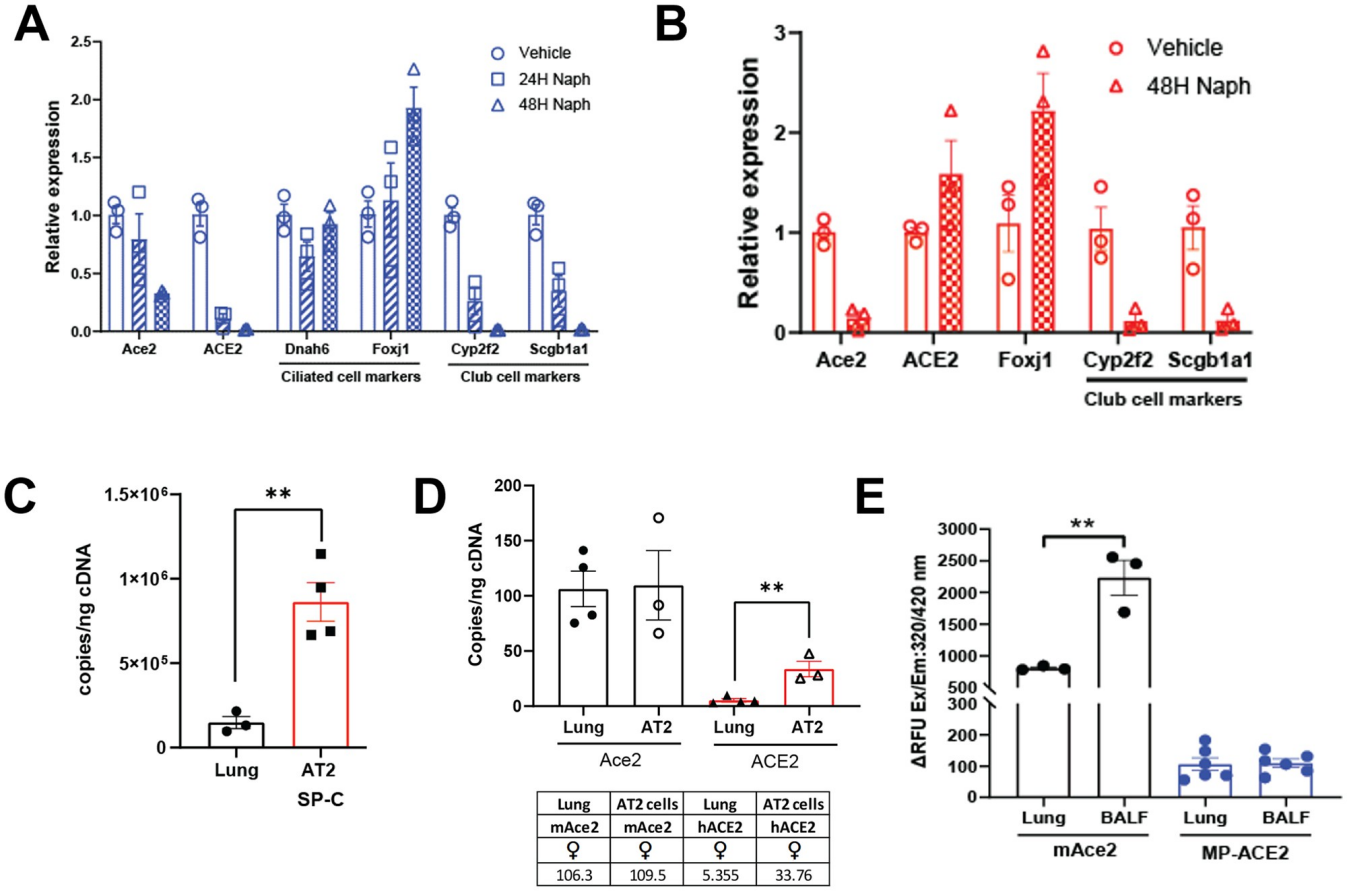
Expression of ACE2 in club and alveolar type II (AT2) cells. **A**. Expression of *ACE2* and *Ace2* in female mice heterozygous for mAce2/MP-ACE2 24 and 48 hours after exposure to naphthalene (Naph) normalized to expression of *ACE2* and *Ace2* in the vehicle treated animals. Club and ciliated cell resistance to naphthalene was evaluated by measuring change in expression of cilia and club cell specific marker genes in samples during this same time interval. **B**. *ACE2* expression is unaltered in heterozygous mAce2/hACE2 treated with naphthalene. **C**. Expression of *Sftpc* verifies enrichment of AT2 population from lung of mAce2/hACE2 heterozygous females. **D**. *ACE2* expression is increased six fold by in airway epithelium enriched for AT2 cells. **E**. Comparison of ACE2 carboxypeptidase activity in lung lysates and BALF of mAce2 and MP-ACE2 mice. For group sizes and statistical comparison between all groups in **E** see S2 Table. ** p<0.01.

A possible explanation for the low expression of *ACE2* in the lung of the MP-ACE2 line is that the human gene is not expressed in club cells, which have been shown to be vulnerable to infection by the SARS-CoV-2 MA10 virus [56]. The sensitivity of mouse club cells to naphthalene because of their unique expression of *Cyp2f2* provided a means of addressing this question [57]. Furthermore, carrying out the study with heterozygous female mice that carry one copy of *Ace2* and one copy of the MP-ACE2 allowed assessment of expression from the human and mouse genes in the same tissue sample. Mice were treated for either 24 or 48 hours with naphthalene after which mRNA prepared from the lungs was evaluated for selective loss of club cells and expression of *Ace2* and *ACE2* was determined (Fig 5A) [58]. Within 48 hours, expression of two club cell mRNAs, *Cyp2f2* (Cytochrome P450 2F2) and *Scgb1a1* (Uteroglobin), could no longer be detected. Expression of *Foxj1*, which is expressed primarily in lung ciliated cells, was significantly increased. This is consistent with previous demonstration of the reparative role of ciliated cells, characterized by an increase in *Foxj1* expression, as these cells lose their cilia and undergo squamous cell metaplasia in response to club death and detachment from the basement membrane [59]. Expression of the ciliated cell-specific *Dnah6* (dynein axonemal heavy chain 6), essential in producing force for ciliary beating, is marginally reduced and then restored as the ciliated cells reestablish normal morphology and function. Expression of *Ace2* could be detected 48 hour. after treatment, albeit at approximately 30% of the level observed in vehicle treated animals. In comparison, all MP-ACE2 expression was lost in the naphthalene treated MP-ACE2 animals, suggesting that expression of ACE2 in the MP-ACE2 mice is largely localized to the *Cyp2f2*-expressing, naphthalene sensitive club cell population. Differences in ACE2 expression between the MP-ACE2 and mAce2 mice could reflect the expression of *Ace2* in one or more additional cell populations in the mouse.

Using a similar strategy, we measured *ACE2* expression in female mice heterozygous for endogenous mouse *Ace2* and hACE2 (Fig 5B). While a substantial decrease in the expression of *Ace2* was observed 48 hours after naphthalene treatment, no change in the expression of the *ACE2* was detected. These results indicate that the human and mouse promoters differ markedly in their ability to direct expression of *Ace2*/*ACE2* to specific cell populations in the lung, with the mouse promoter driving high expression in club cells.

To determine the possible source of *ACE2* expression in the hACE2 mice, populations enriched for alveolar type II (AT2) cells were isolated from heterozygous female mice. Enrichment was verified by assessing changes in expression of the AT2 specific transcript, *Sftpc* (Fig 5C). A six fold increase in expression of ACE2 was observed in the AT2 enriched population (Fig 5D). This is consistent with a model in which AT2 cells are the major cell type in which the human promoter drives *ACE2* expression in the mouse lung.

### Shedding of ACE2 into the airway lumen

ACE2 ectodomain shedding has been reported for human airway epithelia [49] and mouse lungs, where it is reported to play a role in regulation of neutrophil influx [60]. More recently, it has been suggested that the shed ectodomain (soluble ACE2) can mediate entry of SARS-CoV-2 into cells lacking *ACE2* expression [61]. Certainly, loss of ACE2 from the cell membrane will impact it’s vulnerability to viral entry [62]. To determine the sensitivity of the human protein to shedding, we measured human ACE2 in the BALF of the humanized mouse line (Fig 5E). Whole lung lavage was carried out on mAce2 and MP-ACE2 mice prior to tissue collection. Carboxypeptidase activity was determined in both the bronchoalveolar lavage fluid (BALF) and lung homogenates. ACE2 enzyme activity was observed in the lavage and lung samples of both animals, indicating that human protein is actively shed by mouse epithelium.

### Expression of the ACE2 isoforms in the humanized mouse line

**Fig 6:**
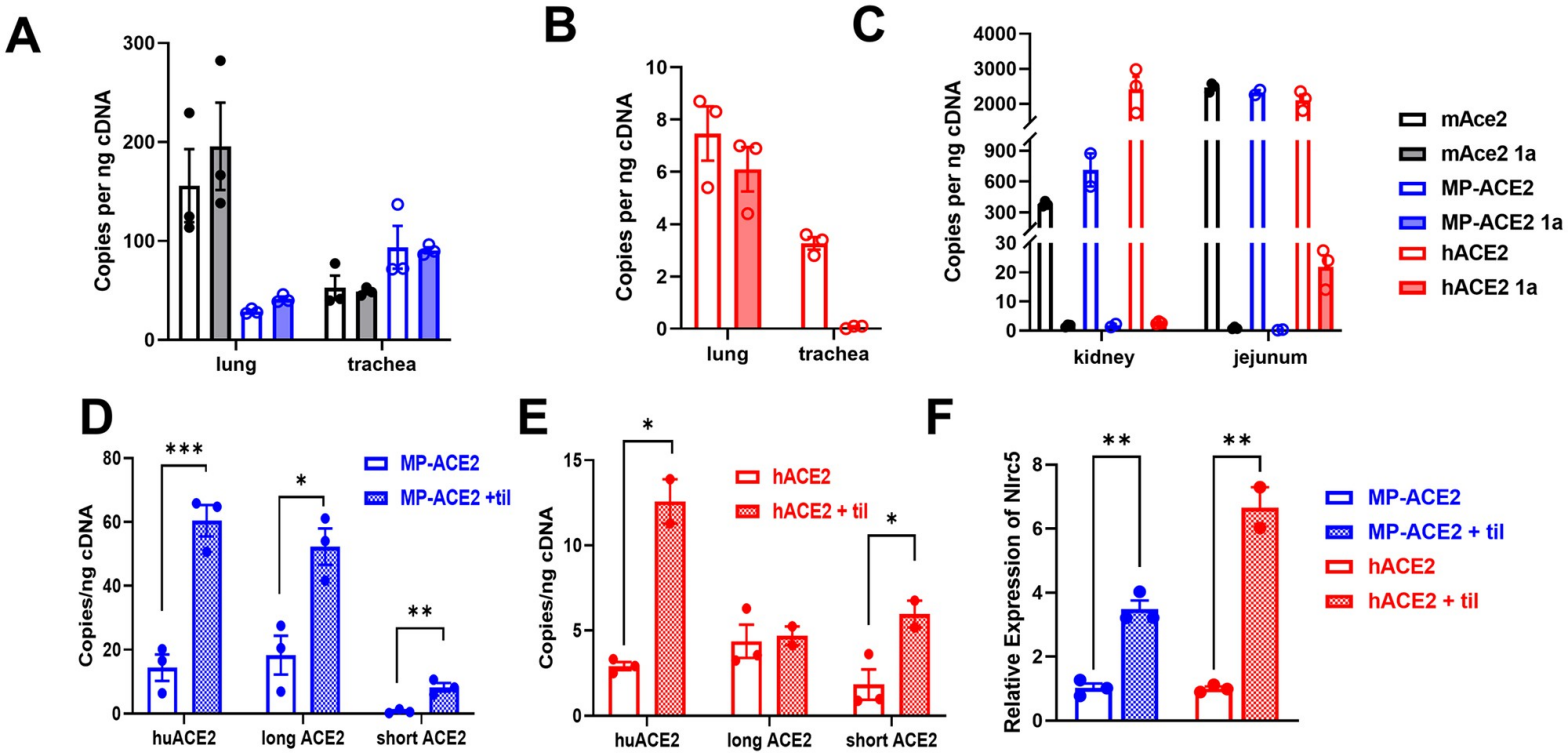
Expression of *ACE2* exon1a and exon1b mRNA transcripts in wild type and humanized mouse lines and induction of exon 1c transcripts after exposure to tilorone. **A-C** Comparison of total *ACE2/Ace2* transcripts measured by ddPCR (open bars) to those that include exon 1a in mRNA (shaded bars) isolated from the tissues and mouse lines indicated. **D-E** shows induction of exon1c transcripts in lung of humanized mice after tilorone (+ til) induced increase in interferon. ddPCR evaluation of *ACE2* lung transcripts using PCR primers that recognized all *ACE2* transcripts (open bars), primers that recognize either the short “Δ” transcript, or primers specific for the “long”, full length *ACE2* mRNA (shaded bars). **F**. Induction of interferon responsive genes (IRG) was verified by demonstration of increased expression of *Nlrc5* gene in the tilorone treated samples by qPCR. Expression is normalized to that measured in vehicle treated animals. For group sizes and statistical comparison between all groups see S1 Table. *p<0.05; ** p<0.01; *** p<0.005.

The *ACE2/Ace2* promoter in both human and mice is bipartite, with full length *ACE2/Ace2* transcripts beginning with one of two initial exons, 1a or 1b [35]. Human RNA-seq studies indicate low levels of exon1a transcripts in most tissue examined, with the highest expression levels reaching approximately 10% of those of exon1b transcripts in the small intestine [63]. Primers specific for transcripts for *ACE2/Ace2* that include exon 1a were generated and used to examine expression of 1a containing transcripts in tissues from the mAce2, MP-ACE2, and hACE2 mice. In mAce2 and MP-ACE2 mice, we found that exon1a containing transcripts comprised all of the transcripts present in the mouse lung and trachea (Fig 6A). In the fully humanized hACE2 mice (Fig 6B), similar to the MP-ACE and mAce2 mice, all the *ACE2* transcripts in the lung included exon1a. However, in contrast to the MP-ACE2 and mAce2 mice, no exon 1a transcripts were observed in the tracheal samples from the hACE2 mice. Thus the human promoter only supports 1b transcripts. In the kidney and intestinal tract, exon1a containing transcripts were absent in all three mouse lines (Fig 6C). This is congruent with the human RNAseq evaluation of exon1a transcripts in that we found about 10% of the transcripts in the intestinal tract of the hACE2 mice included exon 1a [63]. These results indicate that inclusion of exon 1a in transcripts encoding ACE2 varies not only between species but also between tissues. This further supports a model in which expression of *ACE2/Ace2* is regulated in a species- and tissue-specific manner.

### *In vivo* induction of dACE2/short ACE2 by interferon

Evaluation of changes in mRNA expression in airway epithelial cells from naïve and inflamed lungs has identified *ACE2* as an interferon-stimulated gene (ISG) [64,65]. More recently, the effect of interferon on expression of both full length and variant transcripts has been extensively explored in primary epithelium and epithelial cell lines [33,63]. These studies report that interferon mediates the increase in an *ACE2* mRNA transcript initiated in intron 8 from an intronic promoter, which results in the inclusion of an exon unique to primates, exon1c (see Fig 1). The transcript is predicted to encode a truncated ACE2 isoform (short or delta (d)ACE2) that lacks both the carboxypeptidase and SARS-CoV-2 binding domains of the longer protein. However, direct i*n vivo* evidence supporting the induction of *dACE2* expression in response to interferon is lacking, as is evidence for the dependence of its expression on an internal promoter and its specificity for airway epithelium. To this end, the three mouse lines, mAce2, MP-ACE2 and hACE2, were treated with tilorone, a potent interferon inducing drug [66], and expression was evaluated by ddPCR using three sets of primers: primers that recognize all human *ACE2* transcripts, primers that detect only the full length transcript, and primers that detect only the *dACE2* (short *ACE2*) transcript (Fig 6). The induction of ISG in all lines was verified by examination of induction of *Nlrc5* (Fig 6F) [67]. No increase in the expression of *Ace2* was observed in the tilorone treated animals. In contrast, we observed similar fold increases in the copy number of total *ACE2* transcripts in both the MP-ACE2 and the hACE2 lines. The increase in expression of *ACE2* in the fully humanized mouse was accompanied by increases in the *dACE2* but not long *ACE2* transcript. In contrast, an increase in the full length transcript was observed in the MP-ACE2 mice. Thus, while mouse *Ace2* was not induced by interferon, the combined presence of the mouse promoter and the human exon/intron sequences in MP-ACE2 mice rendered the mouse promoter interferon sensitive.

### SARS-CoV-2 infection of MP-ACE2 mice

**Fig 7:**
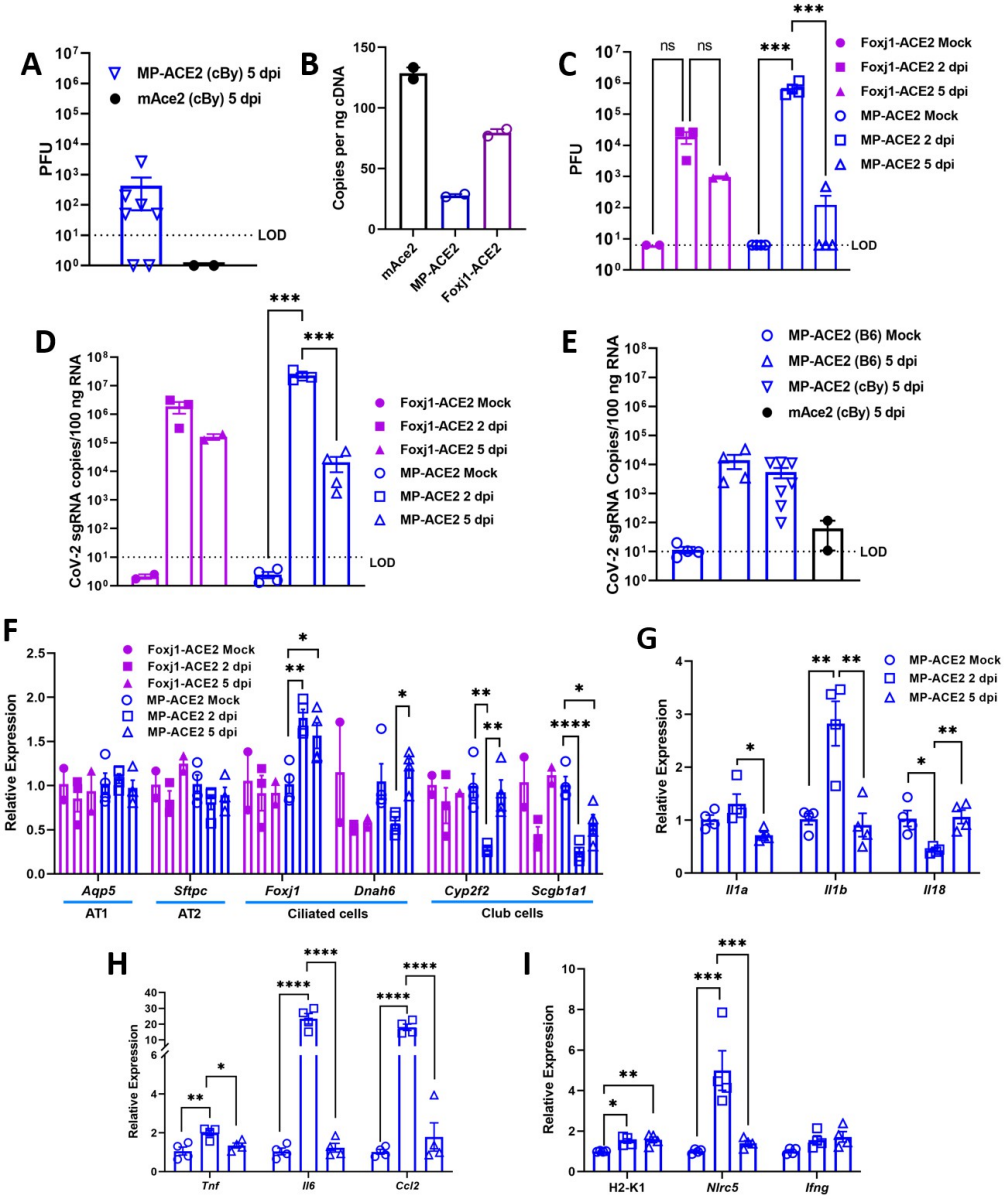
SARS-CoV2 exposure of MP-ACE2, and mAce2 mice. **~**16 week old mice of the indicated genotype were infected with 10^5^ PFUs of SAR-CoV-2 or vehicle (mock infected) and evaluated at either 2 days (DPI 2) or 5 days (DPI 5) for: **A,C** viral titer; **B**, abundance of ACE2 mRNA, determined by ddPCR in FOXJ1-ACE2 transgenic mouse line; **C,D** expression of SARS-CoV-2 nuclear capsid genes; **E**, expression of lung cell-type-specific genes; **D-H**, expression of the indicated pro-inflammatory genes. MP-ACE2 mice were N3/N4 generation C57BL/6N, N2 generation BALB/cBy, or ~N5 generation FOXJ1-ACE2 C57BL/6 (fuchsia). mACE2 (black) are littermates of the BALB/cBy MP-ACE2 animals. For group sizes and statistical comparison between all groups see S1 Table. * p<0.05; ** p<0.01; *** p<0.005; **** p<0.001.

To determine the susceptibility of the humanized lines to SARS-CoV-2 infections, seven MP-ACE2 (N2 BALB/cBy) male mice, 16 weeks of age, and two mAce2 littermates were exposed intranasally (i.n.) to 10^5^ pfu of virus (Fig 7A). No difference in the overall health of the mice was observed over the 5-day period, including no significant change in weight. At the end of this time, animals were euthanized, and homogenates were generated from the lung for evaluation of viral load and for isolation of mRNA. Virus was detected in five of the seven MP-ACE2 mice, although titers were relatively low compared to those achieved with the mouse-adapted virus [56] in all but one of the animals (Fig 7A). As expected, viral titers were below detection in the mAce2 littermate controls. As MP-ACE2 mice are generated by syntenic replacement, they do not express the mouse ACE2 receptor, and thus cell entry is mediated by human ACE2.

To verify that expression of ACE2 in the MP-ACE2 mouse line was sufficient to support viral proliferation, an additional experiment was carried out using MP-ACE2 mice on the C57BL/6N genetic background. In this case, C57BL/6N mice carrying the FOXJ1-ACE2 transgene were included as a positive control for infection and replication of SARS-CoV-2 in the lung. This mouse line, which has been used extensively for study of SARS-CoV and SARS-CoV-2 [27,29,68] carries a transgene in which human *ACE2* expression is driven by the promoter of *FOXJ1*, a gene which in the lung is expressed by ciliated cells [69]. We verified expression of *ACE2* in this line, comparing expression to that in MP-ACE2 mice and to *Ace2* in wild type mice (Fig 7B). Robust expression was observed in the FOXJ1-ACE2 lung, intermediate between that of *Ace2* in mAce2 mice and *ACE2* in MP-ACE2 mice. Because of the rapid resolution of infection in the MP-ACE2 BALB/cBy mice, the lung SARS-CoV-2 load was examined 2 and 5 days after intranasal delivery of 1 x 10^5^ PFU. Mock infected FOXJ1-ACE2 and MP-ACE2 mice were included as controls. Again, no loss of weight was observed in either mock or viral infected MP-ACE2 mice. Despite the lower expression of *ACE2* in the MP-ACE2 mice compared to the FOXJ1-ACE2 animals, the viral loads measured in the lung of the MP-ACE2 were approximately 20-fold higher than those of the FOXJ1-ACE2 mice 2 days post infection. However, because of the small group size, the difference did not achieve significance (Fig 7C). Interestingly, despite the observed higher titers of virus in the MP-ACE2 mice compared to the FOXJ1-ACE2 line 2 days after inoculation, by day 5 the MP-ACE2 mice had cleared the virus while measurable titers remained in the FOXJ1-ACE2 animals.

mRNA was prepared from the lungs of the infected and control animals, and the levels of SARS-CoV-2 subgenomic and genomic nucleocapsid (N) RNA were determined (Fig 7D and 7E). Two days after infection of MP-ACE2 mice, high levels of N-RNA were observed, and, consistent with differences in viral load, these were 10-fold higher in the MP-ACE2 mice compared to the FOXJ1-ACE2 animals. At day 5, despite clearance of virus, high levels of N-RNA were still easily detected. N-RNA was also detected in all 7 MP-ACE2 BALB/cBy mice. As expected, given the species specificity of SARS-CoV-2, nuclear capsid RNA was not detected in RNA prepared from the lungs of the two mAce2 littermates exposed to virus.

### Host response to viral infection

We determined whether SARS-CoV-2 infection led to measurable changes in major lung epithelial cell populations by qPCR analysis of mRNA prepared from mock and SARS-CoV-2 infected FOXJ1-ACE2 and MP-ACE2 mice using a panel of probes specific for alveolar type I (AT1), AT2, ciliated, or club cells. No changes were observed in the expression of AT1 or AT2 cell markers. The pattern of change in the expression of the two club cell specific genes, *Cyp2f2* and *Scgb1a1* in MP-ACE2 mice followed that observed with naphthalene mediated damage to club cells. Expression was decreased two days post infection, with rapid normalization by day 5. Similarly, changes in *Foxj1* expression followed those observed in mice after damage to club cells. In contrast, no significant change in the expression of these genes could be measured in the FOXJ1-ACE2 mice. However, we cannot rule out the possibility that this reflects the smaller number of these mice included in the experiment.

An analysis of the expression of cytokines and chemokines in the infected MP-ACE2 mice revealed a robust immune response to SARS-CoV-2 infection, after which homeostasis was rapidly restored. A significant increase in *Il1b* was observed at day 2, with normalization by day 5 (Fig 7G). *Tnf* and *Ccl2* followed a similar pattern, with elevated expression on day 2 that normalized with reduction of the viral load by day 5 (Fig 7H). A dramatic increase in *Il6*, which was found to be increased six-fold relative to mock infected animals, was detected at day 2 (Fig 7G). While transcripts for *Infg* were more abundant in the SARS-CoV-2 exposed animals at day 2, the increase in expression did not achieve significance. However, a robust and significant increase in the INF-γ sensitive *Nlrc5* gene was observed (Fig 7H). As NLRC5 plays a key role in increased MHC class I gene expression secondary to interferon induction, we examined the expression of *H2-K1* in the lung. A significant increase was observed in expression of this MHC antigen, an important component of both natural killer and cytotoxic T cell mediated removal of virally infected cells [70,71].

**Fig 8:**
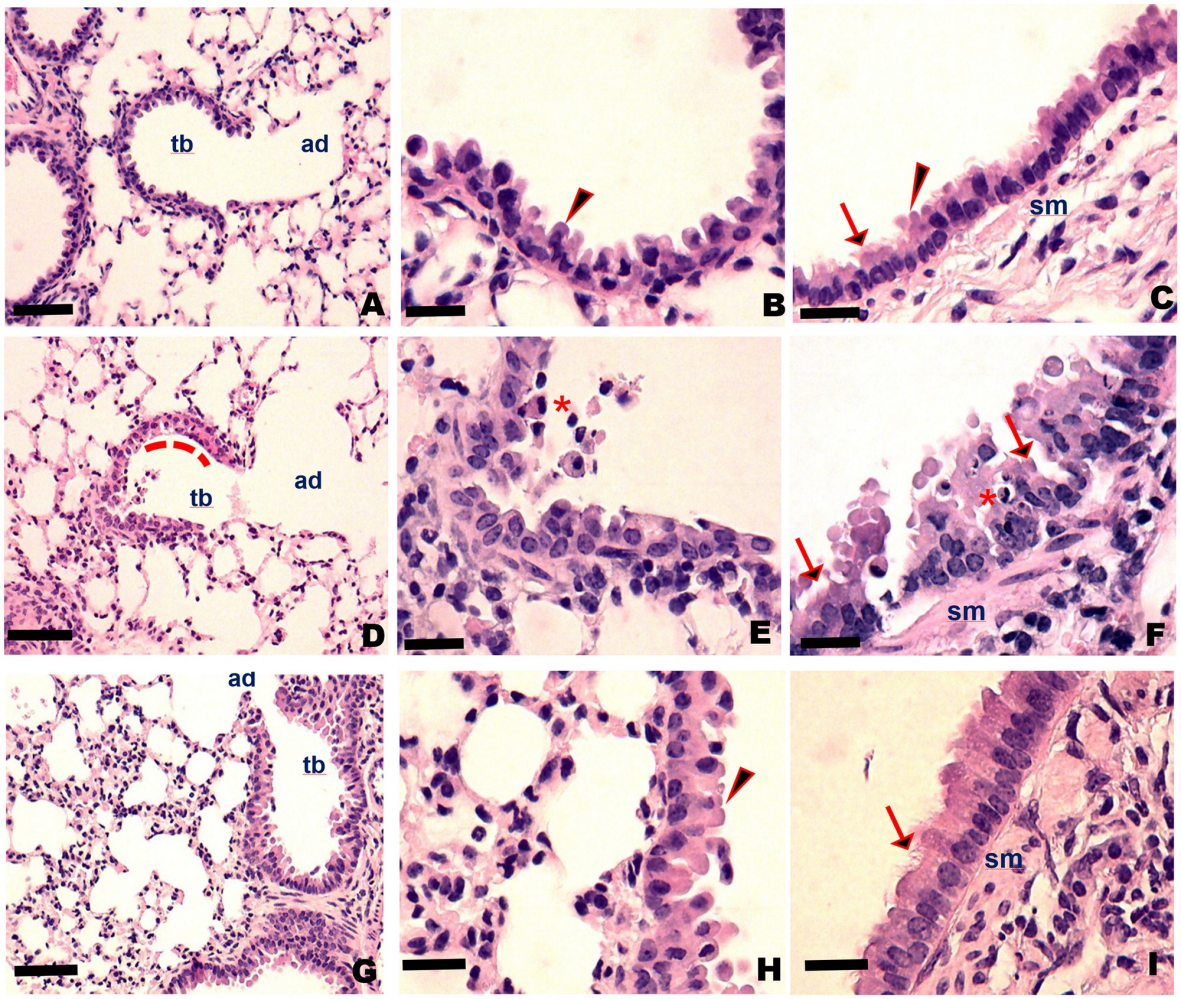
Histological analysis of Foxj1 and MP-ACE2 SARS-CoV-2 infected lungs. Tissue sections from left lobe of lungs from mock-infected and SARS-CoV-2 infected animals at 2 days post infection, fixed and stained with hematoxylin and eosin. (A-C) Mock infected MP-ACE2. (D-F) MP-ACE2 infected animals. (G-I) Foxj1-ACE2 infected animals. (A) Distal lung of a mock infected MP-ACE2 mouse showing a terminal bronchiole (**tb**) and the associated alveolar duct (**ad**) with club cells dominating the epithelium. (B) High magnification of the epithelium showing healthy club cells (**arrowhead**), which are easily identified by eosinophilic “club” shaped cytoplasmic protrusions into the airway. (C) Bronchi of mock-infected animal shows an epithelium dominated by ciliated cells (**arrow**) and submucosal smooth muscle (**sm**) and connective tissue (**ct**) characteristic of larger conducting airways. (D-F) Similar regions of lungs from MP-ACE2 SARS-CoV-2 infected mice show limited changes in architecture of the alveolar/capillary units. However, extensive damage to epithelium (particularly apoptosis/necrosis of club cells), is apparent extending from the terminal bronchiole to the bronchus. (D) Detached cells form a loosely adherent layer of cellular debris at the apical surface of the terminal bronchiole (**red dashed line**). Higher magnification of the epithelium throughout the airway (E,F) shows an abundance of detached epithelial cells with pyknotic nuclei and karyorrhexis, as well as cells with condensed eosinophilic cytoplasm consistent with apoptotic death (**red star**). (G-I) Limited damage to airway epithelium, club cells and ciliated cells was observed in the Foxj1-ACE2 mice. However, changes in the architecture of the distal lung is apparent (G, H) with loss of alveolar/capillary units, increased cellularity and septal thickening. (A,D,G) Bars represent 150 μm. Bars in remaining panels represent 50 μm.

Histological evaluation of the lungs of the MP-ACE2 (Fig 8) mice supports a model in which viral replication in club cells leads to cell death and exfoliation of this airway epithelial population. The distal airways, the alveoli, and alveolar duct remained relatively unaffected. In contrast, loss of club cells as well as morphological changes in club cells consistent with both apoptosis and necrosis were seen throughout the length of the airway (Fig 8D–8F). However, repair was rapid, with little cell death, and repopulation of the airways was observed by 5 days post infection. In contrast, death and exfoliation of airway epithelial cells was less apparent in the Foxj-ACE2 mice, suggesting perhaps an increased tolerance to viral replication. Loss of ciliated cells was not apparent on examination of the larger airways where ciliated cells can be easily identified (Fig 8I). Surprisingly and perhaps in response to failure to clear the virus, histological changes were apparent in the distal lung, with septal thickening, increased cellularity and changes in the architecture of the alveolar unit.

### Differential immune response of the Foxj1-ACE2 and MP-ACE2 mice to viral infection

**Fig 9:**
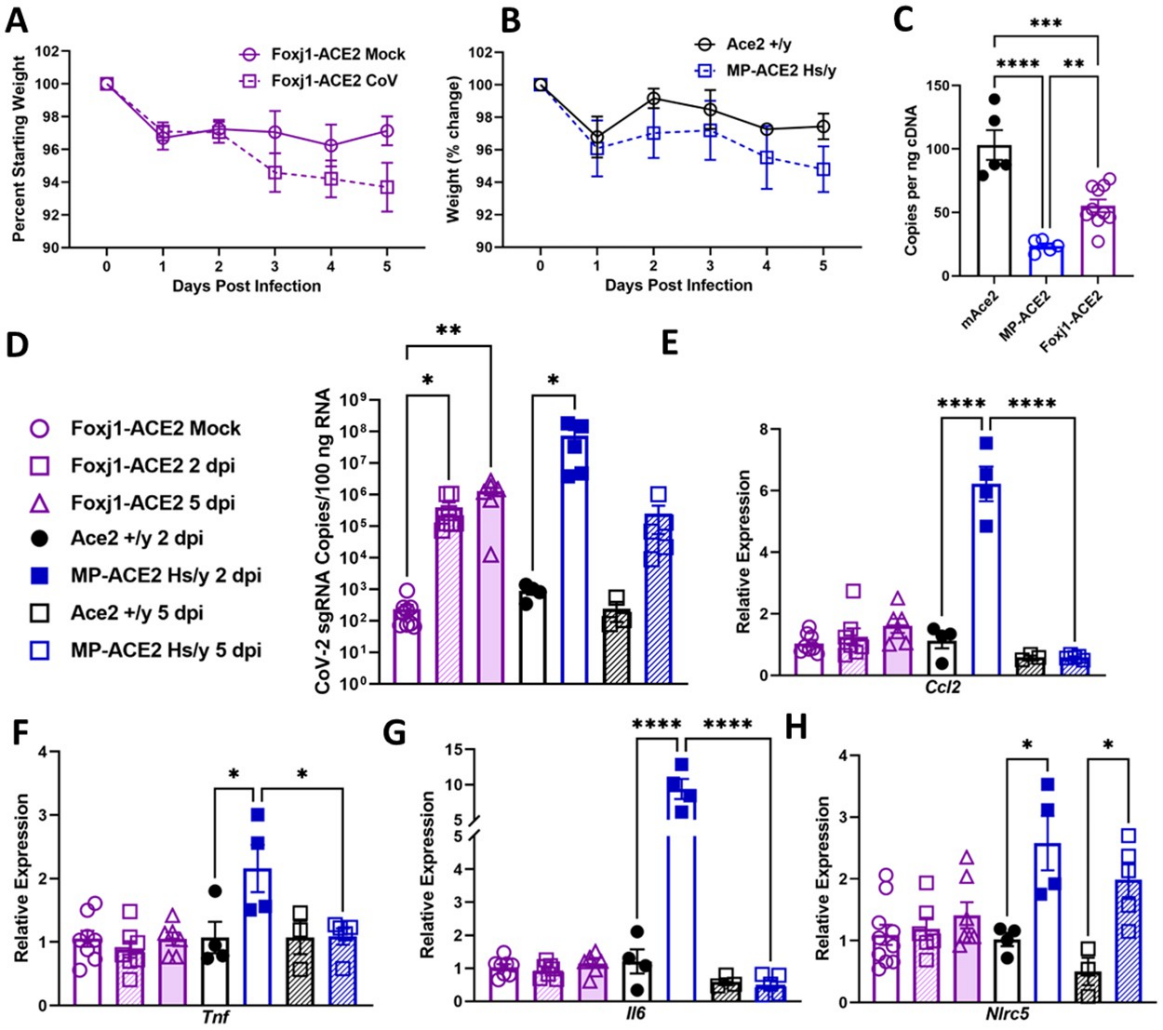
Comparison of SARS-CoV-2 clearance and immune response after exposure of Foxj1-ACE2 and MP-ACE2 mice to SARS-CoV-2. (A and B) Weight loss of mice over the 5 day duration of the experiment did not differ (2 way ANOVA). (C) Expression of ACE2 determined by ddPCR in mice of the indicated genotypes. (D) Copies of SARS-CoV-2 sgRNA (nucleocapsid transcript) present in RNA prepared from the right inferior lobe. (E-H) Expression of the indicated proinflammatory gene. The level of expression in the control animals (mock or mACE2) is assigned a value of one. For MP-ACE2 mice, mAce2 littermates served as control animals, with both receiving an inoculum 10^5^ PFU SARS-CoV-2. For the Foxj1-ACE2 mice, mock (PBS) treated mice served as controls. For group sizes and statistical comparison between all groups, see S1 Table. * p<0.05; ** p<0.01; *** p<0.005; **** p<0.001.

An additional experiment was carried out to verify the robust proliferation of SARS-CoV-2 in the MP-ACE2 mice. A cohort of Foxj1-ACE2 mice was included to further examine the initial observation of differences between the two lines in the clearance of virus over the 5-day period following viral exposure (Fig 9). Mice were infected with 1 x 10^5^ PFUs of virus and weighed daily. A small drop in body weight was observed during the course of the experiment but failed to reach significance for either mouse line relative to mock infected animals (Fig 9A and 9B). No change in body condition score was noted in the mice over the course of the experiment, and the gross appearance of SARS-CoV-2 infected lungs did not differ from those of mock infected animals. Consistent with studies of the smaller Foxj1-ACE2 cohort shown in Fig 7, ACE2 levels in the Foxj1-ACE2 mice were over 2 fold higher than those measured in the MP-ACE2 animals.

Cohorts of animals were euthanized at two days and five days post infection, and ACE2 expression and viral load were assessed by measurement of nucelocapsid sgRNA transcripts present in mRNA prepared from the lung. Surprisingly but consistent with the results described above, a two log drop in sgRNA SARS-CoV-2 levels was observed in the MP-ACE2 mice, while sgRNA levels remained unaltered in the Foxj1-ACE2 animals five days post infection. A major difference in the pathogenesis of disease in the two ACE2 mouse lines was also apparent on evaluation of cytokine levels in the lung. As shown in the previous experiment, the clearance of virus in the MP-ACE2 mice was paralleled by a robust increase in transcripts for Ccl2, TNFα, and IL-6 (Fig 9E–9G). Elevated levels of *Nlrc5* transcripts were observed in the MP-ACE2 mice, indicative of an interferon-mediated increase in expression of MHC antigens. In contrast, no significant increase in the levels of these markers was observed in the Foxj1-ACE2 mice. These changes in gene expression as well as the histological changes we observed on analysis of the MP-ACE2 infected mice are consistent with a model in which SARS-CoV-2 infection of lung ACE2 expressing club cells in the MP-ACE2 mice induces a rapid and protective immune response that limits viral spread. Together, the immune response and death of the infected club cells mediate clearance of virus from the lung and initiation of epithelial repair by 5 days post infection.

## Discussion

To begin to address the complexity of human and mouse ACE2 expression *in vivo*, with a particular focus on the expression pattern in the airways, and to determine how this complexity impacts viral disease mediated by ACE2 dependent coronaviruses, we have generated two mouse lines in which the endogenous *Ace2* locus is humanized by syntenic replacement. These lines differ only in the upstream promoter driving expression of the human gene, with the MP-ACE2 line including the upstream region from the mouse gene and the hACE2 line including the upstream region from the human gene. While no one model can recapitulate all aspects of COVID, we believe that the mouse lines we have generated will provide a unique tool for studying disease pathogenesis and immune responses. In particular, they will be invaluable in understanding how variation in SARS-CoV-2 impacts its ability to circumvent immune surveillance. We therefore believe that these lines represent an improvement relative to previously reported approaches for the study of SARS-CoV-2 pathogenesis in the mouse.

The mouse ACE2 models generated to date fall into two general categories. The first category consists of transgenic models in which expression of a human ACE2 transgene is driven by an exogenous promoter. In these models, the expression pattern is determined largely by the promoter chosen, although it can be modified by the copy number of the transgene and the genomic location or locations into which the transgene has inserted. It is not uncommon for the transgene to form rolling circles prior to insertion, resulting in integration of potentially hundreds of copies into the genome. It is also important to note that, in transgenic models of this type, the endogenous mouse Ace2 gene is still expressed. While this is not a complicating factor for coronaviruses that bind specifically to the human ACE2 protein, some viruses such as Omicron have been reported also to bind mouse ACE2.

The K18-ACE2 model, in which expression of human ACE2 is driven by the promoter of the human KRT18 gene, is the most widely used of the transgenic models [28]. It expresses high levels of human ACE2 receptors on many epithelial cell populations, both in the lung and other organs. The pattern of ACE2 expression observed in this model does not recapitulate that observed in human or in mouse. Infection of mice carrying the K18-ACE2 transgene with SARS-CoV or SARS-CoV2 results in devastating destruction of the lung parenchyma, loss of lung barrier function, and viremia [72]. The severity of the disease observed in this model has made it extremely valuable for the study of vaccines, antibodies that prevent infection, and drugs that limit proliferation of the virus. However, this model cannot recapitulate the pathophysiology and immunopathology of COVID, an infection limited to the upper and conducting airways.

We have included in our results a comparison of our new models to a second transgenic model in which expression of human ACE2 is driven by the promoter from the human FOXJ1 gene [29]. This model differs from the K18-ACE2 transgenic model in that expression of human ACE2 in the lung is primarily limited to ciliated cells, a population of epithelial cells limited to upper airways and conducting airways. Expression of ACE2 in human ciliated cells is well documented.

The two newly developed mouse lines that we describe here fall into a second category of models generated by modification of the endogenous mouse Ace2 locus. Several previously described lines also fall into this category [30,73]. The best described of these models are lines in which a human cDNA has been knocked into the second exon of the mouse locus, so that expression of human ACE2 is regulated by the mouse Ace2 promoter. These lines differ from our MP-ACE2 line in several important ways. First, although normal expression of the mouse Ace2 gene is disrupted in these lines by insertion of the human cDNA, virtually the entire mouse Ace2 gene, including all regulatory elements and coding sequences is still present in the endogenous locus. Second, because the introduced human coding sequence consists of a cDNA, it lacks any intronic regulatory elements present in the full-length human gene and is incapable of giving rise to the short form of ACE2. And third, the introduced human gene includes an exogenous polyadenylation signal which may influence its expression pattern in ways that are difficult to predict. Infection of the ACE2 knock in lines with SARS-CoV results in a milder, non-fatal disease phenotype. Expression of ACE2 in the airways of one of these models has been shown to occur predominantly in club cells [76]. A direct comparison of these knock in lines with our MP-ACE2 line should be helpful in further defining the regulatory impact of intragenic regulatory elements on ACE2 expression in both healthy animals and various disease states.

The previously described ACE2 knock in lines differ from our hACE2 line in that expression of human ACE2 in the hACE2 line is driven by the human ACE2 promoter. Of particular significance, similar to the expression pattern reported for human lung, we did not observe ACE2 expression in the club cells of hACE2 mice. This indicates that expression of ACE2 in this cell type is specific to mouse. Our hACE2 mice will thus provide a more authentic system than the previously described cDNA knock in models for examining the impact of environmental and genetic factors on the activity of the human gene. For example, it should be possible to determine the impact of COVID comorbidities on the activity of the human ACE2 promoter in the conducting airways and lung parenchyma.

A complementary approach that has been taken to allow the use of the mouse as a model system for SARS-CoV-2 research has been the adaptation of the human virus to the mouse. Several mouse-adapted viruses have been generated [56,74]. A major advantage of this approach is that it allows the plethora of mouse knockout lines and mouse disease models to be applied immediately to the study of this respiratory coronavirus. However, compared to the use of mice humanized by syntenic replacement, there are several limitations inherent in this strategy.

First, in addition to changes engineered into the coding sequence of the S protein to facilitate binding of the modified virus to mouse ACE2, creation of an adapted virus capable of causing severe disease required experimental evolution in vivo via serial passage of SARS-CoV-2 MA in the lungs of young adult mice. This process resulted in the introduction of non-synonymous mutations in four other viral genes including in the replicase (NSP4, NSP7, NSP8) and in accessory ORF6 [56], in addition to the changes introduced through reverse genetics into the gene encoding the S protein [68]. It is not unlikely that these changes impact immune detection, innate immune modulation, and aspects of disease pathogenesis. Defining such differences will be important for establishing the translational value of studies using mouse adapted viral strains. As SARS-CoV-2 MA10 still binds human ACE2, this could be accomplished using the lines we describe here.

A second limitation in the use of mouse adapted SARS-CoV-2 is that the approach is of limited usefulness in the study of disease and immune responses to the SARS-CoV-2 variants that will undoubtedly continue to emerge. It is reasonable to assume that these will show variable ability to bind ACE2 of different species. Some of these, such as Omicron, may display some ability to engage the mouse ACE2 receptor [75]. Direct comparison of the SARS-CoV-2 variants will require a model that lacks mouse ACE2 and expresses human ACE2 in relevant cells in the airways, such as those described here.

A final drawback to the use of mouse adapted SARS-CoV-2 is that although the expression pattern of ACE2 is generally similar in mouse and human, as indicated by the present study as well as previous reports, major species differences are also apparent. The lung stands out among those organs displaying the most remarkable quantitative and qualitative differences in ACE2 expression. In contrast to the human airways, in which ACE2 expression diminishes in the proximal to distal direction, extremely high levels of ACE2 are found in the distal mouse lung. As discussed further below, it is not unlikely that the generally higher expression of ACE2 in the mouse lung, as well as differences in the distribution of expression among cell types, impacts the progression of disease and the role played in it by different arms of the immune response. For example, most natural immunity to SARS-CoV-2 in the human population evolved without recruitment of immune populations in response to catastrophic lung damage and loss of barrier function subsequent to infection of the distal lung. Therefore, the usefulness of any mouse adapted virus will be limited by the fact that its ability to bind host cells will be determined by the murine rather than human pattern of ACE2 expression.

Overall, patterns of *ACE2* expression driven by the *hACE2* locus corresponded relatively well to expectations based on previous reports, including recent single-cell RNA-Seq studies. Notable differences were observed between the expression of the human and the mouse genes. Interestingly these differences in expression pattern could not be assigned entirely to the 5’ regulatory region of the gene, as the expression pattern from the *MP-ACE2* locus differed from that of the endogenous mouse *Ace2* locus. This supports a role for motifs within the coding region of the gene in determining gene expression. This model is consistent with the mild SARS-CoV-2 induced disease observed in mice in which the human cDNA coding segment was “knocked in” to the mouse locus, placing expression under the control of the mouse promoter [30,76]. Our studies show that cooperation between regionally unique regulatory elements in the coding region of the gene extend to a number of aspects of gene expression, including the initiation of transcription from exon 1a, 1b, or 1c; the ability of interferon to induce expression of the full length versus the dACE2 (short) transcript; and the extent of sexual dimorphism in ACE2 expression. Furthermore, in the absence of intronic murine regulatory elements, upstream murine regulatory elements alone were insufficient to direct the mouse pattern of expression in the lung.

Our studies also address the extensively discussed vulnerability of the lung to SARS-CoV-2 in light of extremely low expression of the viral receptor in this organ. The lack of a clear consensus on ACE2 expression in the respiratory tract in part reflects the lack of well characterized and validated anti-ACE2 antibodies suitable for immunohistochemistry, which has hampered identification of ACE2-expressing cell populations in the respiratory tract [77]. We chose ab15348 anti-ACE2 rabbit serum for our studies, not only because it recognizes mouse and human ACE2, but also because it has been extensively used to assess ACE2 levels in human lung and airways by immunohistochemistry [78]. Although we were able to visualize human ACE2 protein by western analysis of intestine and kidney using the ab15348 serum, attempts to visualize either mouse or human ACE2 protein in the lung using this approach were unsuccessful. Furthermore, we observed high levels of non-specific hybridization of this antibody with other proteins in both kidney and intestinal lysates. While this lack of specificity may not be observed in all lots of this antibody, it provides an explanation for inconsistencies between reports on the expression of ACE2 in human tissue samples, and thus may explain some of the difficulties in assigning expression to epithelial cell populations by immunohistochemistry. Antibodies directed to SARS-CoV-2 nucleocapsid protein have also been used as a marker for expression of ACE2 [56] in infected mice, in human lung tissue, and in primary cells. Although this approach is convenient because of the high specificity of the antibodies, these reagents provide only surrogate markers for ACE2 and therefore fall short of providing direct evidence of protein expression. In quantifying ACE2 expression in our humanized lines, we therefore chose to rely on ddPCR/qPCR analysis and, when possible, on measurement of ACE2 enzymatic activity.

In our analysis we found that, not only was ACE2 expressed at higher levels in the wildtype mouse lung than in the lungs of the humanized lines, but the pattern of expression differed substantially between wildtype and humanized mice. The high expression of *Ace2* in mouse lung is consistent with the severe disease observed on infection of mice with the mouse adapted SARS-CoV-2 MA10. In contrast, severe lung disease is observed in only a small percentage of humans and is often associated with co- morbidities, including obesity, hypertension, and type II diabetes. Based on single-cell RNA-seq analysis in the human lung and airways, it has been reported previously that *ACE2* is primarily expressed by ciliated cells, although expression by AT2 cells has also been well documented in the healthy lung, where 1% to 3% of these cells express measurable amounts of *ACE2* mRNA. Studies of air-liquid interface cultures established from human lung airways have raised the possibility of an expansion of “tropism” to basal and club cells after initial infection of ciliated cells [79]. Whether this occurs during COVID-19 is not known. In contrast, our results indicate that club cells are the primary contributor to mouse lung *Ace2* mRNA, as removal of these cells results in loss of over 75% of the *Ace2* transcripts.

On the other hand, expression of *ACE2* in human club cells has not been consistently observed, and, consistent with this observation, depletion of club cells from the lungs of our hACE2 mice did not result in a measurable change in the levels of human ACE2 mRNA. This species difference in *ACE2* expression by club cells can be attributed primarily to the mouse upstream regulatory elements, as expression of human *ACE2* in the MP-ACE2 mice was eliminated by club cell depletion. The fact that expression of *ACE2* from the MP-ACE2 locus was decreased relative to that from the endogenous mouse locus suggests that, although the expression of *ACE2* in club cells requires the mouse promoter, intronic elements within the mouse *Ace2* gene can further amplify expression in club cells. Our results indicate that the low expression of *ACE2* in the hACE2 mice largely reflects the lack of expression in club cells. Our studies indicate that ACE2 expression in this line can largely be assigned to AT2 cells, as expression levels increased six-fold in samples enriched for this population. In contrast, enrichment for AT2 cells did not increase expression of the mouse *Ace2* gene.

Despite the fact that mACE2 mice express *ACE2* at higher levels than mACE2 mice, only the latter were susceptible to infection with SARS-CoV-2. These results reiterate the finding that even relatively low levels of expression of human ACE2 are sufficient to render cells permissive to infection by SARS-CoV-2 *in vivo* [68]. The increased severity of disease caused by SARS-CoV-2 MA10 in wildtype mice relative to that caused by SARS-CoV-2 in our MP-ACE2 line may be the result of expression of the endogenous ACE2 receptor in AT2 cells of wildtype mice. Club cells may represent a more dispensable cell population in the mouse lung than AT1 or AT2 cells, as suggested by the rapid recovery of the club cell population after naphthalene exposure. In comparison, treatment with agents such as paraquat, which damage AT2 and AT1 cells, results in long term damage to the lung characterized by fibrosis and loss of alveolar units.

The decrease in expression of two club cell specific mRNAs suggests that the expression profile of club cells is radically altered during viral infection and/or that, similar to their fate after naphthalene exposure, these cells are shed into the airway lumen as part of a well-defined repair process [59]. In contrast, we observed no decrease in expression of ciliated cell markers (*Foxj1*/*Dnah6*) in the lungs of the infected FOXJ1-ACE2 transgenic animals, suggesting that infection of these cells with SARS-CoV-2 may result in a very different sequence of events. Interestingly, although ACE2 expression, measured both by ddPCR and ACE2 activity, was higher in the FOXJ1-ACE2 mice than in the MP-ACE2 mice, the viral titers achieved were approximately ten-fold lower. There are a number of possible explanations for this, including differences in expression of TMRPSS on ciliated and club cells and differences in expression of other proteins required for successful co-opting of the cellular machinery by SARS-CoV-2 while avoiding triggering of immune related pathways. Despite the higher viral load after club cell infection, the lung of the MP-ACE2 mice quickly cleared the virus, while significant titers remained in the ciliated cells of infected FOXJ1-ACE2 mice. This suggests that the cell types infected by SARS-CoV-2 may influence the duration of viral production, epithelial cell death, and the types of cytokines released, thus shaping the nature of the immune response provoked by viral invasion of the lung. Examination of mRNA from the infected MP-ACE2 lungs identified effector molecules that may have a central role in what may be viewed as a protective immune response. Support for the early protective role of some cytokines has been described. For example, IL-6 has been shown to contribute to resolution of RSV infection in mice [80].

SARS-CoV viruses are distinguished from a number of related coronaviruses that cause mild disease by their use of ACE2 as the receptor by which they gain entry into cells. The expression of *ACE2* therefore is an important factor in defining the tropism of the virus, and this, together with other characteristics of ACE2 expressing cells, directs disease pathogenesis. Our studies indicate that the regulation of *ACE2/Ace2* expression is complex, with expression regulated differently in humans and mice as well as within individual tissues within each species. This is particularly true of ACE2 expression in the lung. An understanding of the regulatory mechanisms underlying these differences as well as the impact these differences have on various lung epithelial cell populations will be essential in improving our understanding of the factors underlying COVID-19 pathogenesis.

## Methods

### Ethics and biosafety

All animal work meets the standards of the Institutional Animal Care and Use Committee at University of North Carolina at Chapel Hill as set out in guidelines outlined by the U.S. Department of Agriculture as well as by the Association for the Assessment and Accreditation of Laboratory Animal Care. Safety conditions and approved standard operating procedures have been followed for all experiments involving SARS-CoV-2. BSL3 facilities at University of North Carolina at Chapel Hill were designed to meet safety requirements recommended by the U.S. Department of Health and Human Services, Biosafety in Microbiological and Biomedical Laboratories (BMBL), the National Institutes of Health (NIH), the Centers for Disease Control and Prevention (CDC), and the Public Health Service. We have submitted laboratory safety plans, and the CDC and the University of North Carolina at Chapel Hill Department of Environmental Health and Safety (EHS) have approved the facility for use.

### Generation of mouse lines

The *Ace2* displacer constructs were assembled using a standard recombineering approach. The mouse arms of homology were derived from the 129s7/AB2.2 bMQ BAC library clone bMQ306a09 (Source BioScience). The segment of human genomic DNA containing the *ACE2* gene was derived from the human tile path BAC RP11-478H11 (BACPAC Resources). The resistance marker gene used for selection of embryonic stem (ES) cells in which the Displacer construct underwent genomic integration consisted of a PGK-neo cassette flanked by mutant loxP sites. Excision of the marker gene with transient expression of Cre leaves a nonfunctional lox site in its place. The recombination events for generation of both lines were carried out in the 129 ES cell line, Phnx43. ES cells used were derived from 129SvEv mice. ES cells carrying the correctly modified locus were used to generate chimeric mice, which were bred to 129S6/SvEv/Tac mice (Taconic Bioscience) to maintain the mutation on this genetic background. Chimeras were also bred to C57BL/6NCrl (Charles River Laboratories) and BALC/cBy, strain 000650 (The Jackson Laboratory) mice, and offspring were used for examination of susceptibility to SARS-CoV-2. The MP-ACE2 mouse line is designated by the MMRC as 069708-UNC, 069709-UNC and 069713-UNC; the hACE2 line is designated as 069710-UNC, 069714-UNC, and 069715-UNC; and the FOXJ1-ACE2 line is designated as 066719-UNC.

### Enrichment for alveolar type II cells

Alveolar type II cells were isolated as described previously [81–83]. Briefly, lungs were perfused and airways lavaged to remove blood and airways cells, respectively. The lungs were instilled with one mL of 10 U/mL of Dispase II (Roche) in PBS, followed by 0.5 mL of 1% low melting agarose, removed and incubated in 0.5 mL of Dispase II solution for 45 minutes at room temperature. At the end of the incubation period, digested tissue was transferred to 7 mL of DMEM supplemented with 10% FBS and 0.01% DNase I, teased free of bronchi and bronchioles and further incubated. The resulting cell suspension was filtered through a 70 μm centrifuged at 300 x g for 10 minutes at 4°C. The cell pellet was resuspended in 1 mL of 4% isotonic Percoll (Sigma, St. Louis, MO) and the suspension overlaid on a Percoll step and centrifuged at 400 x g for 20 minutes at 4°C with no brake. AT2 enriched cells were collected at the 10–30% interface and washed once with PBS. To further remove contaminants, cells were stained with biotinylated antibodies against lineage markers (anti-CD45, anti-CD16/32, anti-CD31, and anti-integrin B4), labeled with streptavidin microbeads (Miltenyi Biotec, Bergisch Gladbach, Germany), and then subjected to magnetic separation according to manufacturer’s instructions. The enriched type II cells were collected and used for isolation of RNA.

### RNA preparation and expression analysis

Tissues were collected from male and female mice between 10 and 16 weeks of age. Total RNA was isolated using an RNA isolation solvent (Stat-60; Tel-Test, Friendswood, TX, USA) according to the manufacturer’s protocol. For qRT-PCR, RNA was reverse transcribed to cDNA using a high-capacity cDNA archive kit (Applied Biosystems) following the manufacturer’s recommended protocol. All probes and primers were purchased from a commercial vendor (Applied Biosystems). Amplification of DNA was carried out on the Applied Biosystems 7900 HT Fast RT-PCR System using qBio Blue (Genesee Scientific). Each sample was run in duplicate, and relative expression was determined by normalizing samples to 18S RNA (ΔΔCT). Expression of SARS-CoV-2 nucleocapsid in the lung was determines using the 2019-nCOV_N1 primers, and probes were as follows: Forward primer: GACCCC AAA ATC AGC GAA AT, Reverse primer: TCT GGT TAC TGC CAG TTG AAT CTG, Probe: AC CCC GCA TT ACG TTT GGT GGA CC (CDC N1 qRT-PCR assay) [84]. Comparison was to a CoV-2 standard curve RNA produced by PCR amplification of SARS-CoV-2 nucleocapsid by which a 5’ T7 polymerase promoter was introduced. This amplicon was used as template to generate in vitro transcribed RNA, which was then quantified and serially diluted (10^8^–10^1^ copies/μl).

### Droplet digital PCR (ddPCR)

RNA and cDNA were prepared as described above. ddPCR reactions were performed with QX200 Droplet Digital PCR System (Bio-Rad, Hercules, CA) according to the manufacturer’s instructions. Each FAM reaction mixture (20 μL) contained 10 μL ddPCR Supermix for Probes (No dUTP) (Bio-Rad), 9 μL of template cDNA (up 100 ng), and 1 μL of Primers/Probes (900 nM per primer and 250 nM probe). And SYBR Green 20 μL reaction mixtures contained 10 μL iTaq Universal SYBR Green Supermix (Bio-Rad), 1 μL of primers (250 nM each primer), and 9 μL of template cDNA (up to 100 ng). The reaction mixtures were loaded into a disposable droplet generator cartridge (Bio-Rad), and droplets were formed using Bio-Rad QX-100 emulsification device. The contents were transferred to a 96-well Eppendorf reaction plate (Genesee Scientific; Morrisville, NC) and sealed with foil using Eppendorf 96-well heat sealer. PCR amplification of the droplets was performed using a C1000 Touch Thermal Cycler (Bio-Rad) with the following parameters: 95°C for 10 min, followed by 40 cycles of 94°C for 30 sec and 60°C for 1 min, and a final 98°C for 10 min. After PCR amplification, the plate was scanned using a QX200 Droplet Reader (Bio-Rad). QX Manager Software (Bio-Rad) was used to analyze the data by calculating the absolute copy number of the target DNA (units of copies/μL) using Poisson distribution analysis. All TaqMan gene expression probes were purchased from Applied Biosystems. Primers for SYBR reactions were designed using the NCBI Primer Designing Tool.

### ACE2 activity in tissues, urine and bronchial lavage fluid

ACE2 activity in tissue lysates was measured using specific fluorogenic ACE2 substrate (Mca-APK-(Dnp) (AnaSpec, San Jose, CA) in the presence or absence of the ACE2 inhibitor (MLN-4760) (Sigma-Aldrich, St. Louis, MO) as previously described [85]. Tissue samples were homogenized in lysis buffer (75 mM Tris-HCl, pH 7.5, 1 M NaCl, 0.5 mM ZnCl2, 0.01 mM Captopril, 0.1 mM Z-Pro-Prolinal, 1mM PMSF, EDTA-free inhibitor cocktail tablet from Roche, and 0.5% Triton X-100) and centrifuged at 14,000 x g for 10 minutes at 4°C. Protein concentration in tissue lysates was measured using the Bradford method. Tissue lysates (10 μg of protein for kidney extracts and 40 μg of protein for heart, lung, trachea, and sinus extracts) were pre incubated with 70 μL of assay buffer (75 mM Tris-HCl, pH 7.5, 1 M NaCl, 0.5 mM ZnCl2, 0.01 mM Captopril, 0.1 mM Z-Pro-Prolinal, and EDTA-free inhibitor cocktail tablet from Roche) with or without ACE inhibitor MLN-4760 (10 μM final) for 30 minutes at room temperature. After the incubation with ACE2 inhibitor, 30 μL of ACE2 substrate buffer (75 mM Tris-HCl, pH 7.5, 1 M NaCl, 0.5 mM ZnCl2, 0.01 mM Captopril, 0.1 mM Z-Pro-Prolinal, and 0.167 mM Mca-APK-Dpn) was added to each well to initiate the reaction. Samples were incubated in the dark for 1 hour at room temperature, and fluorescence values were measured at an excitation wavelength of 320 nm and emission wavelength of 420 nm using a BioTek Cytation5 plate reader (BioTek instruments, Winooski, VT). Results were expressed as ΔRFU (Relative Fluorescence Unit) after subtraction of RFU values obtained in the presence of MLN-4760. BALF was collected from mice as previously described, and urine was collected during necropsy.

### Naphthalene treatment

Mice received a single intraperitoneal injection of naphthalene (Sigma-Aldrich, St. Louis, MO) solution (200 mg/kg) freshly prepared in corn oil. As controls, mice were injected with vehicle only. Mice were euthanized at 24 or 48 hours after the injections. Lungs were then surgically removed, snap frozen, and stored at -80°C for subsequent RNA extraction.

### Virus strains

The virus strains icSARS-CoV-2 WT [86] and SARS-CoV-2 WT stocks were grown using Vero E6 cells and titered via plaque assay. Vero E6 cells were cultured in Dulbecco’s modified Eagle’s medium (DMEM, GIBCO), 5% Fetal Clone II serum (Hyclone), and 1X antibiotic/antimycotic (GIBCO). Briefly, serially diluted virus was added to a monolayer of Vero E6 cells and overlayed with media containing 0.8% agarose. Plaques were counted after three days after visualization by staining with Neutral Red dye. All viral infections were carried out under biosafety level 3 (BSL-3) conditions under negative pressure. All personnel conducting viral experiments wore Tyvek suits equipped with personal powered air-purifying respirators.

### *In Vivo* infection

All mice were bred at UNC at Chapel Hill. Anesthetized (ketamine/xylazine) mice were intranasally infected with 1 x 10^5^ PFU of SARS-CoV-2, whereas mock infected mice received only PBS. Mice were monitored daily for any weight loss or decrease in lung function. For determination of viral load and RNA analysis, samples were collected at indicated time points after euthanizing mice by isoflurane overdose.

### Histology

After euthanasia, the left lung lobe was harvested inflated with 10% phosphate buffered formalin and then further fixed by submersion in fixative for 7 days. Lung lobes were embedded in paraffin and sectioned at 3 μm thickness. Sequential sections were stained with hematoxylin and eosin.

### Quantification and statistical analysis

Build-in functions of GraphPad Prism were used for data analyses and visualization. Specific statistical tests as well as numbers of animals are included in respective figure legends and detailed in S1–S3 Tables. These include the number of mice in each group and comparisons between all mice of each sex and genotype.

## Supporting information

S1 Fig Expression of Ace2/ACE2 in jejunum from male and female offspring of wild type female mice bred to chimeras generated with MP-ACE2 ES cells. The ES cell line used in these studies was male, and thus, as the Ace2 locus is on the X chromosome, female offspring were expected to inherit one copy of the humanized locus from the chimeric male mouse and one copy of the endogenous murine locus from the wild type dam. Due to random X inactivation, approximately 50% of cells in female offspring were expected to express human ACE2 from the *MP-ACE2* locus gene and approximately 50% were expected to express mouse Ace2 from the endogenous *Ace2* locus. Consistent with this expectation, ddPCR analysis of mRNA prepared from the female offspring confirmed expression from both the endogenous *Ace2* locus and the *MP-ACE2* locus. In contrast, because male mice inherited their Y chromosome from the ES cell chimera and their X chromosome from the wild type dam, they were expected to express only the mouse *Ace2* gene. Consistent with this expectation, expression of the *MP-ACE2* locus was not detected in male offspring, confirming the species specificity of the probe set used in these studies. Each dot represents an individual animal. **** p<0.001.
(TIF)

S2 Fig Comparison of expression and activity of ACE2 in cardiac tissue of the humanized mouse lines. **A**. ddPCR evaluation of *ACE2/Ace2* copies present in cDNA prepared from heart tissue of male (♂) and female (♀) mice of the indicated genotype. Mean value for male and females of each genotype is shown below the bar graph. For comparison, expression level of *Ace* in male and females mice is shown at the left. Following intestine, kidney, testis, and gallbladder, the highest levels of ACE2 expression have been reported in human heart tissue [52]. *Ace2* expression was easily detected by ddPCR in whole heart from mAce2 mice, albeit at approximately 30-fold lower levels than those measured in the kidney Levels were also approximately 30-fold lower than those of *Ace*, and the dramatic sexual dimorphism characteristic of *Ace* expression was not as apparent on comparison of *Ace2* mRNA levels between males and females [86]. Exchange of the mouse coding introns/exons for those of the human gene resulted in a major reduction in expression, with a 10-fold decrease in *ACE2* mRNA levels in the MP-ACE2 mice. Again, this difference was surprising given that the expression of the human gene was directed by the mouse promoter. Higher expression of the human gene, approaching that in mice, was observed when the locus was fully humanized. **B**. ACE2 activity measured in tissue lysates from mice of the indicated sex and genotype. Activity was easily observed in the whole heart homogenates prepared from mAce2 mice, and, consistent with the mRNA analysis levels, enzyme activity was extremely low in samples from MP-ACE2 animals. Despite very similar RNA levels, the enzyme activity in the samples from the fully humanized hACE2 mice was reduced approximately 4 fold. However, the activity remained measurably higher than that observed in the MP-ACE2 lines. For group sizes and statistical comparison between all groups in, **A** and **B**, see S1–S3 Tables.
(TIF)

S3 Fig Enrichment for respiratory and olfactory epithelium. Epithelial tissue was removed during necropsy from female mice heterozygous for the human and mouse Ace2 locus based on anatomical location. The identity of the samples was verified by assessing the relative expression of *Muc5ac*, which is expressed by goblet cells in the respiratory epithelium and mouse gene encoding UGT2A1/2 which is expressed by the olfactory epithelium.
(TIF)

S1 Table Statistical analysis for Figs 2B, 3A, 4A, 4C, 4E, 6A, 6B, 6C, 6D, 6E, 6F, 7A, 7B, 7C, 7D, 7E, 7F, 7G, 7H, 7I, 9C, 9D, 9E, 9F, 9G and 9H, and S1–S3 Figs. (XLSX)

S2 Table Statistical analysis of ACE activity levels. (XLSX)

S3 Table Primers used for quantitative PCR. (XLSX)

## References

[1] Murgolo N, Therien AG, Howell B, Klein D, Koeplinger K, et al. SARS-CoV-2 tropism, entry, replication, and propagation: Considerations for drug discovery and development. PLoS Pathog (2021) 17: e1009225. doi: 10.1371/journal.ppat.1009225 33596266 PMC7888651

[2] Jackson CB, Farzan M, Chen B, Choe H Mechanisms of SARS-CoV-2 entry into cells. Nature Reviews Molecular Cell Biology (2022) 23: 3–20. doi: 10.1038/s41580-021-00418-x 34611326 PMC8491763

[3] Puray-Chavez M, LaPak KM, Schrank TP, Elliott JL, Bhatt DP, et al. Systematic analysis of SARS-CoV-2 infection of an ACE2-negative human airway cell. Cell Rep (2021) 36: 109364. doi: 10.1016/j.celrep.2021.109364 34214467 PMC8220945

[4] Hoffmann M, Kleine-Weber H, Schroeder S, Kruger N, Herrler T, et al. SARS-CoV-2 Cell Entry Depends on ACE2 and TMPRSS2 and Is Blocked by a Clinically Proven Protease Inhibitor. Cell (2020) 181: 271–280 e278. doi: 10.1016/j.cell.2020.02.052 32142651 PMC7102627

[5] Li W, Moore MJ, Vasilieva N, Sui J, Wong SK, et al. Angiotensin-converting enzyme 2 is a functional receptor for the SARS coronavirus. Nature (2003) 426: 450–454. doi: 10.1038/nature02145 14647384 PMC7095016

[6] Hofmann H, Pyrc K, van der Hoek L, Geier M, Berkhout B, et al. Human coronavirus NL63 employs the severe acute respiratory syndrome coronavirus receptor for cellular entry. Proc Natl Acad Sci U S A (2005) 102: 7988–7993. doi: 10.1073/pnas.0409465102 15897467 PMC1142358

[7] Shulla A, Heald-Sargent T, Subramanya G, Zhao J, Perlman S, et al. A transmembrane serine protease is linked to the severe acute respiratory syndrome coronavirus receptor and activates virus entry. J Virol (2011) 85: 873–882. doi: 10.1128/JVI.02062-10 21068237 PMC3020023

[8] Heurich A, Hofmann-Winkler H, Gierer S, Liepold T, Jahn O, et al. TMPRSS2 and ADAM17 cleave ACE2 differentially and only proteolysis by TMPRSS2 augments entry driven by the severe acute respiratory syndrome coronavirus spike protein. J Virol (2014) 88: 1293–1307. doi: 10.1128/JVI.02202-13 24227843 PMC3911672

[9] Donoghue M, Hsieh F, Baronas E, Godbout K, Gosselin M, et al. A novel angiotensin-converting enzyme-related carboxypeptidase (ACE2) converts angiotensin I to angiotensin 1–9. Circ Res (2000) 87: E1–9. doi: 10.1161/01.res.87.5.e1 10969042

[10] Turner AJ, Hooper NM The angiotensin-converting enzyme gene family: genomics and pharmacology. Trends Pharmacol Sci (2002) 23: 177–183. doi: 10.1016/s0165-6147(00)01994-5 11931993

[11] Camargo SM, Singer D, Makrides V, Huggel K, Pos KM, et al. Tissue-specific amino acid transporter partners ACE2 and collectrin differentially interact with hartnup mutations. Gastroenterology (2009) 136: 872–882. doi: 10.1053/j.gastro.2008.10.055 19185582 PMC7094282

[12] Hashimoto T, Perlot T, Rehman A, Trichereau J, Ishiguro H, et al. ACE2 links amino acid malnutrition to microbial ecology and intestinal inflammation. Nature (2012) 487: 477–481. doi: 10.1038/nature11228 22837003 PMC7095315

[13] Singer D, Camargo SM, Ramadan T, Schafer M, Mariotta L, et al. Defective intestinal amino acid absorption in Ace2 null mice. Am J Physiol Gastrointest Liver Physiol (2012) 303: G686–695. doi: 10.1152/ajpgi.00140.2012 22790597

[14] Alenina N, Bader M ACE2 in Brain Physiology and Pathophysiology: Evidence from Transgenic Animal Models. Neurochem Res (2019) 44: 1323–1329. doi: 10.1007/s11064-018-2679-4 30443713 PMC7089194

[15] Guy JL, Jackson RM, Acharya KR, Sturrock ED, Hooper NM, et al. Angiotensin-converting enzyme-2 (ACE2): comparative modeling of the active site, specificity requirements, and chloride dependence. Biochemistry (2003) 42: 13185–13192. doi: 10.1021/bi035268s 14609329

[16] Serfozo P, Wysocki J, Gulua G, Schulze A, Ye M, et al. Ang II (Angiotensin II) Conversion to Angiotensin-(1–7) in the Circulation Is POP (Prolyloligopeptidase)-Dependent and ACE2 (Angiotensin-Converting Enzyme 2)-Independent. Hypertension (2020) 75: 173–182. doi: 10.1161/HYPERTENSIONAHA.119.14071 31786979 PMC7286421

[17] Iwai M, Horiuchi M Devil and angel in the renin-angiotensin system: ACE-angiotensin II-AT1 receptor axis vs. ACE2-angiotensin-(1–7)-Mas receptor axis. Hypertens Res (2009) 32: 533–536. doi: 10.1038/hr.2009.74 19461648 PMC7091931

[18] Kragstrup TW, Singh HS, Grundberg I, Nielsen AL, Rivellese F, et al. Plasma ACE2 predicts outcome of COVID-19 in hospitalized patients. PLoS One (2021) 16: e0252799. doi: 10.1371/journal.pone.0252799 34086837 PMC8177449

[19] Imai Y, Kuba K, Rao S, Huan Y, Guo F, et al. Angiotensin-converting enzyme 2 protects from severe acute lung failure. Nature (2005) 436: 112–116. doi: 10.1038/nature03712 16001071 PMC7094998

[20] Zhang H, Baker A Recombinant human ACE2: acing out angiotensin II in ARDS therapy. Crit Care (2017) 21: 305. doi: 10.1186/s13054-017-1882-z 29237475 PMC5729230

[21] Vickers C, Hales P, Kaushik V, Dick L, Gavin J, et al. Hydrolysis of biological peptides by human angiotensin-converting enzyme-related carboxypeptidase. J Biol Chem (2002) 277: 14838–14843. doi: 10.1074/jbc.M200581200 11815627

[22] Sodhi CP, Wohlford-Lenane C, Yamaguchi Y, Prindle T, Fulton WB, et al. Attenuation of pulmonary ACE2 activity impairs inactivation of des-Arg(9) bradykinin/BKB1R axis and facilitates LPS-induced neutrophil infiltration. Am J Physiol Lung Cell Mol Physiol (2018) 314: L17–L31. doi: 10.1152/ajplung.00498.2016 28935640 PMC5866432

[23] Ramos SG, Rattis B, Ottaviani G, Celes MRN, Dias EP ACE2 Down-Regulation May Act as a Transient Molecular Disease Causing RAAS Dysregulation and Tissue Damage in the Microcirculatory Environment Among COVID-19 Patients. Am J Pathol (2021) 191: 1154–1164. doi: 10.1016/j.ajpath.2021.04.010 33964216 PMC8099789

[24] Cousin VL, Giraud R, Bendjelid K Pathophysiology of COVID-19: Everywhere You Look You Will See ACE2! Front Med (Lausanne) (2021) 8: 694029. doi: 10.3389/fmed.2021.694029 34513868 PMC8429613

[25] Tikellis C, Bialkowski K, Pete J, Sheehy K, Su Q, et al. ACE2 deficiency modifies renoprotection afforded by ACE inhibition in experimental diabetes. Diabetes (2008) 57: 1018–1025. doi: 10.2337/db07-1212 18235039

[26] Patel VB, Mori J, McLean BA, Basu R, Das SK, et al. ACE2 Deficiency Worsens Epicardial Adipose Tissue Inflammation and Cardiac Dysfunction in Response to Diet-Induced Obesity. Diabetes (2016) 65: 85–95. doi: 10.2337/db15-0399 26224885 PMC4686955

[27] Jiang RD, Liu MQ, Chen Y, Shan C, Zhou YW, et al. Pathogenesis of SARS-CoV-2 in Transgenic Mice Expressing Human Angiotensin-Converting Enzyme 2. Cell (2020) 182: 50–58 e58. doi: 10.1016/j.cell.2020.05.027 32516571 PMC7241398

[28] McCray PB Jr., Pewe L, Wohlford-Lenane C, Hickey M, Manzel L, et al. Lethal infection of K18-hACE2 mice infected with severe acute respiratory syndrome coronavirus. J Virol (2007) 81: 813–821. doi: 10.1128/JVI.02012-06 17079315 PMC1797474

[29] Menachery VD, Yount BL Jr., Sims AC, Debbink K, Agnihothram SS, et al. SARS-like WIV1-CoV poised for human emergence. Proc Natl Acad Sci U S A (2016) 113: 3048–3053. doi: 10.1073/pnas.1517719113 26976607 PMC4801244

[30] Sun SH, Chen Q, Gu HJ, Yang G, Wang YX, et al. A Mouse Model of SARS-CoV-2 Infection and Pathogenesis. Cell Host Microbe (2020) 28: 124–133 e124. doi: 10.1016/j.chom.2020.05.020 32485164 PMC7250783

[31] Li Y, Cao L, Li G, Cong F, Li Y, et al. Remdesivir Metabolite GS-441524 Effectively Inhibits SARS-CoV-2 Infection in Mouse Models. J Med Chem. (2021) doi: 10.1021/acs.jmedchem.0c01929 33523654

[32] Blaschke RJ, Rappold GA Man to mouse—lessons learned from the distal end of the human X chromosome. Genome Res (1997) 7: 1114–1117. doi: 10.1101/gr.7.12.1114 9414316

[33] Blume C, Jackson CL, Spalluto CM, Legebeke J, Nazlamova L, et al. A novel ACE2 isoform is expressed in human respiratory epithelia and is upregulated in response to interferons and RNA respiratory virus infection. Nat Genet (2021) 53: 205–214. doi: 10.1038/s41588-020-00759-x 33432184

[34] Ye M, Wysocki J, Gonzalez-Pacheco FR, Salem M, Evora K, et al. Murine recombinant angiotensin-converting enzyme 2: effect on angiotensin II-dependent hypertension and distinctive angiotensin-converting enzyme 2 inhibitor characteristics on rodent and human angiotensin-converting enzyme 2. Hypertension (2012) 60: 730–740. doi: 10.1161/HYPERTENSIONAHA.112.198622 22777933 PMC3426447

[35] Pedersen KB, Chhabra KH, Nguyen VK, Xia H, Lazartigues E The transcription factor HNF1alpha induces expression of angiotensin-converting enzyme 2 (ACE2) in pancreatic islets from evolutionarily conserved promoter motifs. Biochim Biophys Acta (2013) 1829: 1225–1235. 24100303 10.1016/j.bbagrm.2013.09.007 PMC3838857

[36] Komatsu T, Suzuki Y, Imai J, Sugano S, Hida M, et al. Molecular cloning, mRNA expression and chromosomal localization of mouse angiotensin-converting enzyme-related carboxypeptidase (mACE2). DNA Seq (2002) 13: 217–220. doi: 10.1080/1042517021000021608 12487024

[37] Wiener RS, Cao YX, Hinds A, Ramirez MI, Williams MC Angiotensin converting enzyme 2 is primarily epithelial and is developmentally regulated in the mouse lung. J Cell Biochem (2007) 101: 1278–1291. doi: 10.1002/jcb.21248 17340620 PMC7166549

[38] Itoyama S, Keicho N, Hijikata M, Quy T, Phi NC, et al. Identification of an alternative 5’-untranslated exon and new polymorphisms of angiotensin-converting enzyme 2 gene: lack of association with SARS in the Vietnamese population. Am J Med Genet A (2005) 136: 52–57. doi: 10.1002/ajmg.a.30779 15937940 PMC7138097

[39] Tukiainen T, Villani AC, Yen A, Rivas MA, Marshall JL, et al. Landscape of X chromosome inactivation across human tissues. Nature (2017) 550: 244–248. doi: 10.1038/nature24265 29022598 PMC5685192

[40] Uri K, Fagyas M, Kertesz A, Borbely A, Jenei C, et al. Circulating ACE2 activity correlates with cardiovascular disease development. J Renin Angiotensin Aldosterone Syst (2016) 17. doi: 10.1177/1470320316668435 27965422 PMC5843890

[41] Pedersen KB, Sriramula S, Chhabra KH, Xia H, Lazartigues E Species-specific inhibitor sensitivity of angiotensin-converting enzyme 2 (ACE2) and its implication for ACE2 activity assays. Am J Physiol Regul Integr Comp Physiol (2011) 301: R1293–1299. doi: 10.1152/ajpregu.00339.2011 21880865 PMC3213941

[42] Nadarajah R, Milagres R, Dilauro M, Gutsol A, Xiao F, et al. Podocyte-specific overexpression of human angiotensin-converting enzyme 2 attenuates diabetic nephropathy in mice. Kidney Int (2012) 82: 292–303. doi: 10.1038/ki.2012.83 22475818 PMC3410252

[43] Chou CF, Loh CB, Foo YK, Shen S, Fielding BC, et al. ACE2 orthologues in non-mammalian vertebrates (Danio, Gallus, Fugu, Tetraodon and Xenopus). Gene (2006) 377: 46–55. doi: 10.1016/j.gene.2006.03.010 16781089 PMC7125734

[44] Mizuiri S, Hemmi H, Arita M, Ohashi Y, Tanaka Y, et al. Expression of ACE and ACE2 in individuals with diabetic kidney disease and healthy controls. Am J Kidney Dis (2008) 51: 613–623. doi: 10.1053/j.ajkd.2007.11.022 18371537

[45] Lely AT, Hamming I, van Goor H, Navis GJ Renal ACE2 expression in human kidney disease. J Pathol (2004) 204: 587–593. doi: 10.1002/path.1670 15538735

[46] Liu J, Ji H, Zheng W, Wu X, Zhu JJ, et al. Sex differences in renal angiotensin converting enzyme 2 (ACE2) activity are 17beta-oestradiol-dependent and sex chromosome-independent. Biol Sex Differ (2010) 1: 6. 21208466 10.1186/2042-6410-1-6 PMC3010099

[47] Wysocki J, Ye M, Soler MJ, Gurley SB, Xiao HD, et al. ACE and ACE2 activity in diabetic mice. Diabetes (2006) 55: 2132–2139. doi: 10.2337/db06-0033 16804085

[48] Lambert DW, Yarski M, Warner FJ, Thornhill P, Parkin ET, et al. Tumor necrosis factor-alpha convertase (ADAM17) mediates regulated ectodomain shedding of the severe-acute respiratory syndrome-coronavirus (SARS-CoV) receptor, angiotensin-converting enzyme-2 (ACE2). J Biol Chem (2005) 280: 30113–30119. doi: 10.1074/jbc.M505111200 15983030 PMC8062222

[49] Jia HP, Look DC, Tan P, Shi L, Hickey M, et al. Ectodomain shedding of angiotensin converting enzyme 2 in human airway epithelia. Am J Physiol Lung Cell Mol Physiol (2009) 297: L84–96. doi: 10.1152/ajplung.00071.2009 19411314 PMC2711803

[50] Warner FJ, Lew RA, Smith AI, Lambert DW, Hooper NM, et al. Angiotensin-converting enzyme 2 (ACE2), but not ACE, is preferentially localized to the apical surface of polarized kidney cells. J Biol Chem (2005) 280: 39353–39362. doi: 10.1074/jbc.M508914200 16166094

[51] Shaltout HA, Westwood BM, Averill DB, Ferrario CM, Figueroa JP, et al. Angiotensin metabolism in renal proximal tubules, urine, and serum of sheep: evidence for ACE2-dependent processing of angiotensin II. Am J Physiol Renal Physiol (2007) 292: F82–91. doi: 10.1152/ajprenal.00139.2006 16896185

[52] Hikmet F, Mear L, Edvinsson A, Micke P, Uhlen M, et al. The protein expression profile of ACE2 in human tissues. Mol Syst Biol (2020) 16: e9610. doi: 10.15252/msb.20209610 32715618 PMC7383091

[53] Nawijn MC, Timens W Can ACE2 expression explain SARS-CoV-2 infection of the respiratory epithelia in COVID-19? Mol Syst Biol (2020) 16: e9841. doi: 10.15252/msb.20209841 32715628 PMC7383087

[54] Jedlitschky G, Cassidy AJ, Sales M, Pratt N, Burchell B Cloning and characterization of a novel human olfactory UDP-glucuronosyltransferase. Biochem J (1999) 340 (Pt 3): 837–843. 10359671 PMC1220318

[55] Amini SE, Gouyer V, Portal C, Gottrand F, Desseyn JL Muc5b is mainly expressed and sialylated in the nasal olfactory epithelium whereas Muc5ac is exclusively expressed and fucosylated in the nasal respiratory epithelium. Histochem Cell Biol (2019) 152: 167–174. doi: 10.1007/s00418-019-01785-5 31030254

[56] Leist SR, Dinnon KH 3rd, Schafer A, Tse LV, Okuda K, et al. A Mouse-Adapted SARS-CoV-2 Induces Acute Lung Injury and Mortality in Standard Laboratory Mice. Cell (2020) 183: 1070–1085 e1012. doi: 10.1016/j.cell.2020.09.050 33031744 PMC7510428

[57] Reid WD, Ilett KF, Glick JM, Krishna G Metabolism and binding of aromatic hydrocarbons in the lung. Relationship to experimental bronchiolar necrosis. Am Rev Respir Dis (1973) 107: 539–551. doi: 10.1164/arrd.1973.107.4.539 4697663

[58] Mahvi D, Bank H, Harley R Morphology of a naphthalene-induced bronchiolar lesion. Am J Pathol (1977) 86: 558–572. 842612 PMC2032128

[59] Park KS, Wells JM, Zorn AM, Wert SE, Laubach VE, et al. Transdifferentiation of ciliated cells during repair of the respiratory epithelium. Am J Respir Cell Mol Biol (2006) 34: 151–157. doi: 10.1165/rcmb.2005-0332OC 16239640 PMC2644179

[60] Sodhi CP, Nguyen J, Yamaguchi Y, Werts AD, Lu P, et al. A Dynamic Variation of Pulmonary ACE2 Is Required to Modulate Neutrophilic Inflammation in Response to Pseudomonas aeruginosa Lung Infection in Mice. J Immunol (2019) 203: 3000–3012. doi: 10.4049/jimmunol.1900579 31645418 PMC7458157

[61] Yeung ML, Teng JLL, Jia L, Zhang C, Huang C, et al. Soluble ACE2-mediated cell entry of SARS-CoV-2 via interaction with proteins related to the renin-angiotensin system. Cell (2021) 184: 2212–2228 e2212. doi: 10.1016/j.cell.2021.02.053 33713620 PMC7923941

[62] Kazemi S, Lopez-Munoz AD, Holly J, Jin L, Yewdell JW, et al. Variations in cell-surface ACE2 levels alter direct binding of SARS-CoV-2 Spike protein and viral infectivity: Implications for measuring Spike protein interactions with animal ACE2 orthologs. bioRxiv. (2021) doi: 10.1101/2021.10.21.465386 36000847 PMC9472623

[63] Onabajo OO, Banday AR, Stanifer ML, Yan W, Obajemu A, et al. Interferons and viruses induce a novel truncated ACE2 isoform and not the full-length SARS-CoV-2 receptor. Nat Genet (2020) 52: 1283–1293. doi: 10.1038/s41588-020-00731-9 33077916 PMC9377523

[64] Ziegler CGK, Allon SJ, Nyquist SK, Mbano IM, Miao VN, et al. SARS-CoV-2 Receptor ACE2 Is an Interferon-Stimulated Gene in Human Airway Epithelial Cells and Is Detected in Specific Cell Subsets across Tissues. Cell (2020) 181: 1016–1035 e1019. doi: 10.1016/j.cell.2020.04.035 32413319 PMC7252096

[65] Chua RL, Lukassen S, Trump S, Hennig BP, Wendisch D, et al. COVID-19 severity correlates with airway epithelium-immune cell interactions identified by single-cell analysis. Nat Biotechnol (2020) 38: 970–979. doi: 10.1038/s41587-020-0602-4 32591762

[66] Stringfellow DA, Glasgow LA Tilorone hydrochloride: an oral interferon-inducing agent. Antimicrob Agents Chemother (1972) 2: 73–78. doi: 10.1128/AAC.2.2.73 4670490 PMC444270

[67] Meissner TB, Li A, Biswas A, Lee KH, Liu YJ, et al. NLR family member NLRC5 is a transcriptional regulator of MHC class I genes. Proc Natl Acad Sci U S A (2010) 107: 13794–13799. doi: 10.1073/pnas.1008684107 20639463 PMC2922274

[68] Dinnon KH 3rd, Leist SR, Schafer A, Edwards CE, Martinez DR, et al. A mouse-adapted model of SARS-CoV-2 to test COVID-19 countermeasures. Nature (2020) 586: 560–566. doi: 10.1038/s41586-020-2708-8 32854108 PMC8034761

[69] Ostrowski LE, Hutchins JR, Zakel K, O’Neal WK Targeting expression of a transgene to the airway surface epithelium using a ciliated cell-specific promoter. Mol Ther (2003) 8: 637–645. doi: 10.1016/s1525-0016(03)00221-1 14529837

[70] Hoglund P, Brodin P Current perspectives of natural killer cell education by MHC class I molecules. Nat Rev Immunol (2010) 10: 724–734. doi: 10.1038/nri2835 20818413

[71] van de Sandt CE, Barcena M, Koster AJ, Kasper J, Kirkpatrick CJ, et al. Human CD8(+) T Cells Damage Noninfected Epithelial Cells during Influenza Virus Infection In Vitro. Am J Respir Cell Mol Biol (2017) 57: 536–546. doi: 10.1165/rcmb.2016-0377OC 28613916

[72] Winkler ES, Bailey AL, Kafai NM, Nair S, McCune BT, et al. SARS-CoV-2 infection of human ACE2-transgenic mice causes severe lung inflammation and impaired function. Nat Immunol (2020) 21: 1327–1335. doi: 10.1038/s41590-020-0778-2 32839612 PMC7578095

[73] Zhou B, Thao TTN, Hoffmann D, Taddeo A, Ebert N, et al. SARS-CoV-2 spike D614G change enhances replication and transmission. Nature (2021) 592: 122–127. doi: 10.1038/s41586-021-03361-1 33636719

[74] Muruato A, Vu MN, Johnson BA, Davis-Gardner ME, Vanderheiden A, et al. Mouse-adapted SARS-CoV-2 protects animals from lethal SARS-CoV challenge. PLoS Biol (2021) 19: e3001284. doi: 10.1371/journal.pbio.3001284 34735434 PMC8594810

[75] Halfmann PJ, Iida S, Iwatsuki-Horimoto K, Maemura T, Kiso M, et al. SARS-CoV-2 Omicron virus causes attenuated disease in mice and hamsters. Nature. (2022) doi: 10.1038/s41586-022-04441-6 35062015 PMC8942849

[76] Winkler ES, Chen RE, Alam F, Yildiz S, Case JB, et al. SARS-CoV-2 Causes Lung Infection without Severe Disease in Human ACE2 Knock-In Mice. J Virol (2022) 96: e0151121. doi: 10.1128/JVI.01511-21 34668780 PMC8754206

[77] Zamorano Cuervo N, Grandvaux N ACE2: Evidence of role as entry receptor for SARS-CoV-2 and implications in comorbidities. Elife (2020) 9. doi: 10.7554/eLife.61390 33164751 PMC7652413

[78] Lee IT, Nakayama T, Wu CT, Goltsev Y, Jiang S, et al. ACE2 localizes to the respiratory cilia and is not increased by ACE inhibitors or ARBs. Nat Commun (2020) 11: 5453. doi: 10.1038/s41467-020-19145-6 33116139 PMC7595232

[79] Ravindra NG, Alfajaro MM, Gasque V, Huston NC, Wan H, et al. Single-cell longitudinal analysis of SARS-CoV-2 infection in human airway epithelium identifies target cells, alterations in gene expression, and cell state changes. PLoS Biol 19: (2021) e3001143. doi: 10.1371/journal.pbio.3001143 33730024 PMC8007021

[80] Pyle CJ, Uwadiae FI, Swieboda DP, Harker JA Early IL-6 signalling promotes IL-27 dependent maturation of regulatory T cells in the lungs and resolution of viral immunopathology. PLoS Pathog (2017) 13: e1006640. doi: 10.1371/journal.ppat.1006640 28953978 PMC5633202

[81] Corti M, Brody AR, Harrison JH Isolation and primary culture of murine alveolar type II cells. Am J Respir Cell Mol Biol (1996) 14: 309–315. doi: 10.1165/ajrcmb.14.4.8600933 8600933

[82] Sinha M, Lowell CA Isolation of Highly Pure Primary Mouse Alveolar Epithelial Type II Cells by Flow Cytometric Cell Sorting. Bio Protoc (2016) 6. doi: 10.21769/BioProtoc.2013 28180137 PMC5293249

[83] Weller NK, Karnovsky MJ Improved isolation of rat lung alveolar type II cells. More representative recovery and retention of cell polarity. Am J Pathol (1986) 122: 92–100. 2934989 PMC1888136

[84] Lu X, Wang L, Sakthivel SK, Whitaker B, Murray J, et al. US CDC Real-Time Reverse Transcription PCR Panel for Detection of Severe Acute Respiratory Syndrome Coronavirus 2. Emerg Infect Dis (2020) 26. doi: 10.3201/eid2608.201246 32396505 PMC7392423

[85] Wang Y, Cassis LA, Thatcher SE Use of a Fluorescent Substrate to Measure ACE2 Activity in the Mouse Abdominal Aorta. Methods Mol Biol (2017) 1614: 61–67. doi: 10.1007/978-1-4939-7030-8_5 28500595 PMC6442458

[86] Hou YJ, Okuda K, Edwards CE, Martinez DR, Asakura T, et al. SARS-CoV-2 Reverse Genetics Reveals a Variable Infection Gradient in the Respiratory Tract. Cell (2020) 182: 429–446 e414. doi: 10.1016/j.cell.2020.05.042 32526206 PMC7250779

