# Supplementary figures and images for: Human ACE2 expression, a major tropism determinant for SARS-CoV-2, is regulated by upstream and intragenic elements
Source: PLoS Pathog. 2023 Feb 22;19(2):e1011168. doi: 10.1371/journal.ppat.1011168 (PMC9987828; doi:10.1371/journal.ppat.1011168)

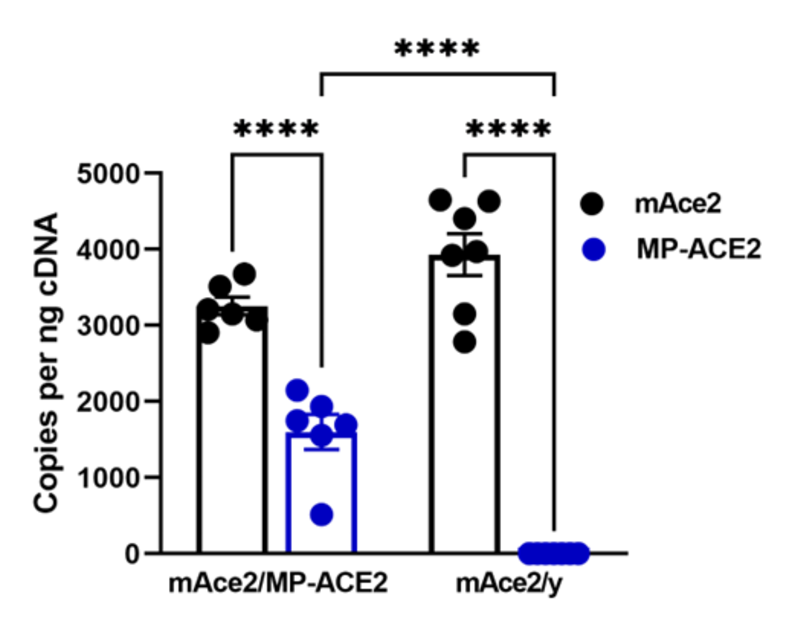

Supplement: S1 Fig — The ES cell line used in these studies was male, and thus, as the Ace2 locus is on the X chromosome, female offspring were expected to inherit one copy of the humanized locus from the chimeric male mouse and one copy of the endogenous murine locus from the wild type dam. Due to random X inactivation, approximately 50% of cells in female offspring were expected to express human ACE2 from the MP-ACE2 locus gene and approximately 50% were expected to express mouse Ace2 from the endogenous Ace2 locus. Consistent with this expectation, ddPCR analysis of mRNA prepared from the female offspring confirmed expression from both the endogenous Ace2 locus and the MP-ACE2 locus. In contrast, because male mice inherited their Y chromosome from the ES cell chimera and their X chromosome from the wild type dam, they were expected to express only the mouse Ace2 gene. Consistent with this expectation, expression of the MP-ACE2 locus was not detected in male offspring, confirming the species specificity of the probe set used in these studies. Each dot represents an individual animal. **** p<0.001. (TIF) [file ppat.1011168.s001.tif]

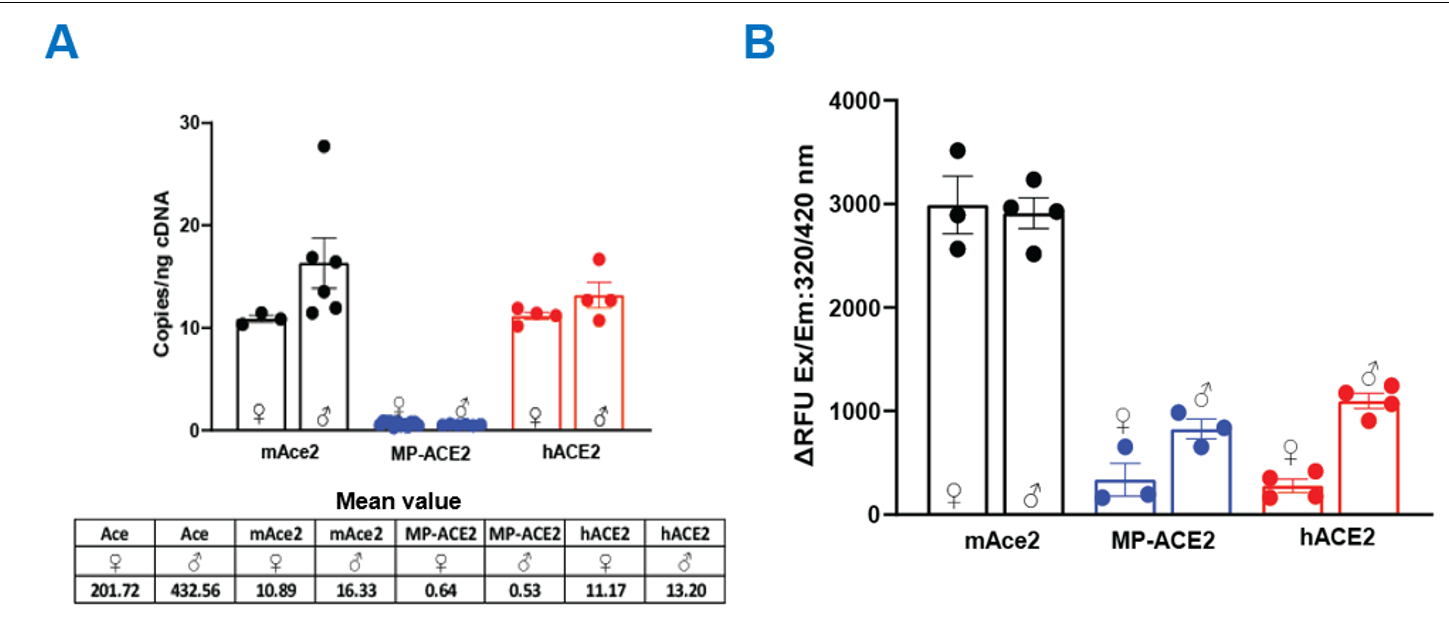

Supplement: S2 Fig — A. ddPCR evaluation of ACE2/Ace2 copies present in cDNA prepared from heart tissue of male (♂) and female (♀) mice of the indicated genotype. Mean value for male and females of each genotype is shown below the bar graph. For comparison, expression level of Ace in male and females mice is shown at the left. Following intestine, kidney, testis, and gallbladder, the highest levels of ACE2 expression have been reported in human heart tissue [52]. Ace2 expression was easily detected by ddPCR in whole heart from mAce2 mice, albeit at approximately 30-fold lower levels than those measured in the kidney Levels were also approximately 30-fold lower than those of Ace, and the dramatic sexual dimorphism characteristic of Ace expression was not as apparent on comparison of Ace2 mRNA levels between males and females [86]. Exchange of the mouse coding introns/exons for those of the human gene resulted in a major reduction in expression, with a 10-fold decrease in ACE2 mRNA levels in the MP-ACE2 mice. Again, this difference was surprising given that the expression of the human gene was directed by the mouse promoter. Higher expression of the human gene, approaching that in mice, was observed when the locus was fully humanized. B. ACE2 activity measured in tissue lysates from mice of the indicated sex and genotype. Activity was easily observed in the whole heart homogenates prepared from mAce2 mice, and, consistent with the mRNA analysis levels, enzyme activity was extremely low in samples from MP-ACE2 animals. Despite very similar RNA levels, the enzyme activity in the samples from the fully humanized hACE2 mice was reduced approximately 4 fold. However, the activity remained measurably higher than that observed in the MP-ACE2 lines. For group sizes and statistical comparison between all groups in, A and B, see S1–S3 Tables. (TIF) [file ppat.1011168.s002.tif]

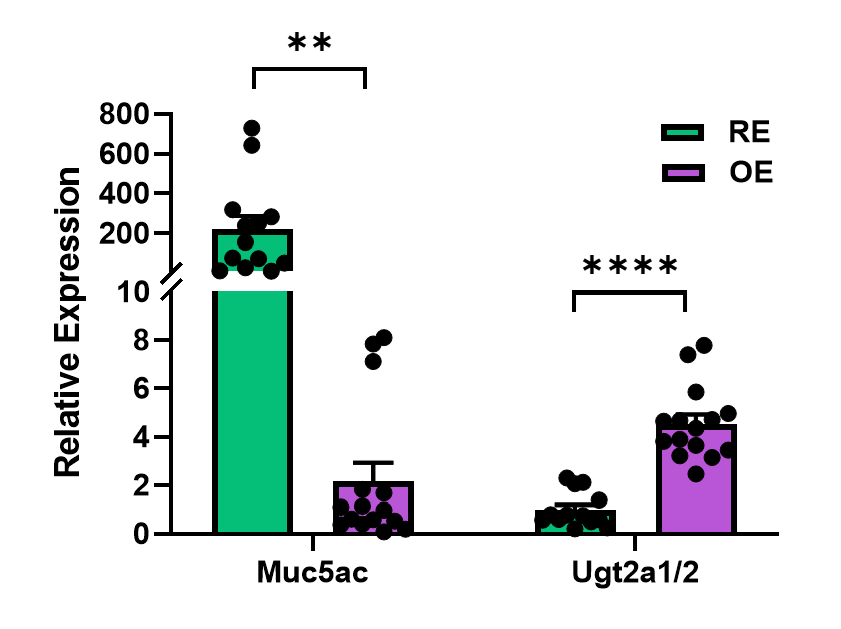

Supplement: S3 Fig — Epithelial tissue was removed during necropsy from female mice heterozygous for the human and mouse Ace2 locus based on anatomical location. The identity of the samples was verified by assessing the relative expression of Muc5ac, which is expressed by goblet cells in the respiratory epithelium and mouse gene encoding UGT2A1/2 which is expressed by the olfactory epithelium. (TIF) [file ppat.1011168.s003.tif]
